# Assessing Metabolism and Injury in Acute Human Traumatic Brain Injury with Magnetic Resonance Spectroscopy: Current and Future Applications

**DOI:** 10.3389/fneur.2017.00426

**Published:** 2017-09-12

**Authors:** Matthew G. Stovell, Jiun-Lin Yan, Alison Sleigh, Marius O. Mada, T. Adrian Carpenter, Peter J. A. Hutchinson, Keri L. H. Carpenter

**Affiliations:** ^1^Division of Neurosurgery, Department of Clinical Neurosciences, University of Cambridge, Cambridge, United Kingdom; ^2^Department of Neurosurgery, Keelung Chang Gung Memorial Hospital, Chang Gung University College of Medicine, Taoyuan, Taiwan; ^3^Wolfson Brain Imaging Centre, Department of Clinical Neurosciences, University of Cambridge, Cambridge, United Kingdom; ^4^National Institute for Health Research/Wellcome Trust Clinical Research Facility, Cambridge University Hospitals NHS Foundation Trust, Cambridge, United Kingdom

**Keywords:** ^1^H MRS, ^31^P MRS, ^13^C MRS, trauma, traumatic brain injury, energy metabolism, biomarker

## Abstract

Traumatic brain injury (TBI) triggers a series of complex pathophysiological processes. These include abnormalities in brain energy metabolism; consequent to reduced tissue pO_2_ arising from ischemia or abnormal tissue oxygen diffusion, or due to a failure of mitochondrial function. *In vivo* magnetic resonance spectroscopy (MRS) allows non-invasive interrogation of brain tissue metabolism in patients with acute brain injury. Nuclei with “spin,” e.g., ^1^H, ^31^P, and ^13^C, are detectable using MRS and are found in metabolites at various stages of energy metabolism, possessing unique signatures due to their chemical shift or spin–spin interactions (J-coupling). The most commonly used clinical MRS technique, ^1^H MRS, uses the great abundance of hydrogen atoms within molecules in brain tissue. Spectra acquired with longer echo-times include *N*-acetylaspartate (NAA), creatine, and choline. NAA, a marker of neuronal mitochondrial activity related to adenosine triphosphate (ATP), is reported to be lower in patients with TBI than healthy controls, and the ratio of NAA/creatine at early time points may correlate with clinical outcome. ^1^H MRS acquired with shorter echo times produces a more complex spectrum, allowing detection of a wider range of metabolites.^31^ P MRS detects high-energy phosphate species, which are the end products of cellular respiration: ATP and phosphocreatine (PCr). ATP is the principal form of chemical energy in living organisms, and PCr is regarded as a readily mobilized reserve for its replenishment during periods of high utilization. The ratios of high-energy phosphates are thought to represent a balance between energy generation, reserve and use in the brain. In addition, the chemical shift difference between inorganic phosphate and PCr enables calculation of intracellular pH.^13^ C MRS detects the ^13^C isotope of carbon in brain metabolites. As the natural abundance of ^13^C is low (1.1%), ^13^C MRS is typically performed following administration of ^13^C-enriched substrates, which permits tracking of the metabolic fate of the infused ^13^C in the brain over time, and calculation of metabolic rates in a range of biochemical pathways, including glycolysis, the tricarboxylic acid cycle, and glutamate–glutamine cycling. The advent of new hyperpolarization techniques to transiently boost signal in ^13^C-enriched MRS *in vivo* studies shows promise in this field, and further developments are expected.

## Introduction

### Metabolic Dysfunction in Traumatic Brain Injury (TBI)

Traumatic brain injury is the commonest cause of death and disability in young adults in the developed world and is a significant demand on resources (1). If a person survives the initial traumatic insult a series of pathophysiological processes occur causing further damage to the brain that results in greater disability and even death. These include raised intracranial pressure (ICP), cerebral hypoperfusion, generalized hypoxia, hypoglycemia, neuroinflammation, and metabolic dysfunction. Metabolic dysfunction describes the brain relying on glycolysis (despite the presence of oxygen) as a rapid but inefficient means of synthesizing adenosine triphosphate (ATP)—so generating much less ATP per mole of glucose consumed than if the pyruvate produced by glycolysis feeds into mitochondrial metabolism. It is often ascribed to a failure of mitochondrial function (2, 3). Due to advances in neurointensive care and multimodality monitoring, gross hypoxia and hypoperfusion are generally avoided in patients, and raised ICP is identified and managed. The monitoring, interpretation and treatment of brain metabolic dysfunction and neuroinflammation are more challenging.

**Figure 1:**
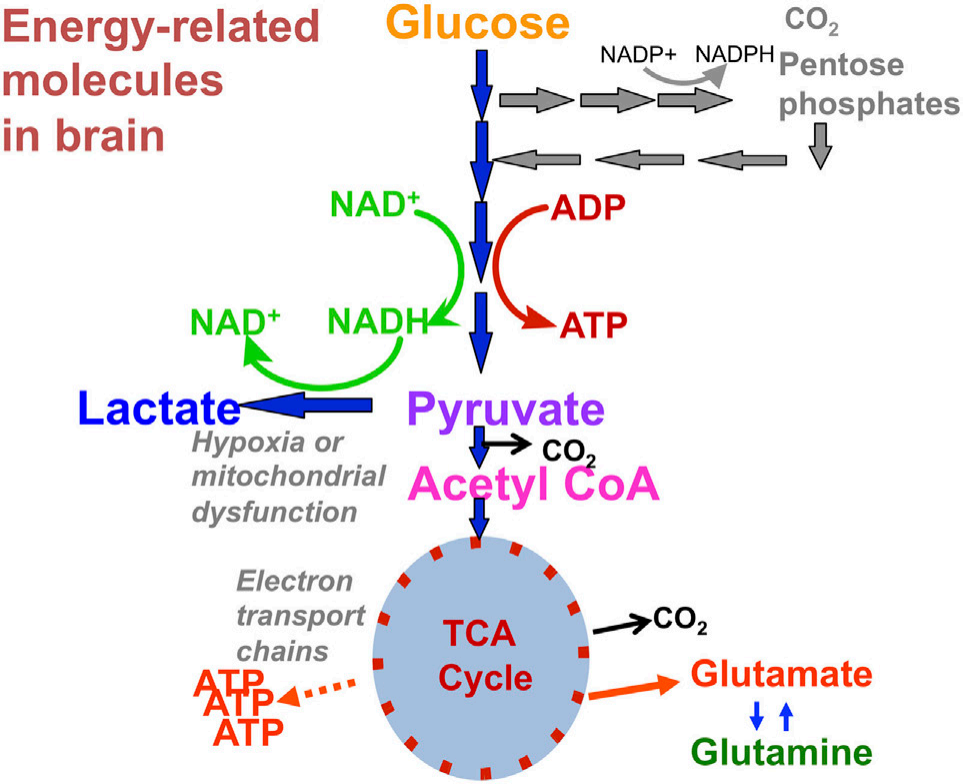
Simplified schematic of major energy pathways in the brain includes glycolysis, which takes place in the cytosol and produces pyruvate, which enters mitochondria and is converted into acetyl-CoA that enters the tricarboxylic acid (TCA) cycle. Alternatively, pyruvate can stay in the cytosol and is converted into lactate that is exported out of the cell. The pentose phosphate pathway takes place in the cytosol and is an alternative pathway that can be upregulated after injury; it is an important source of NADPH used to produce the reduced form of glutathione (GSH) for preventing oxidative stress. This figure was originally published by Carpenter et al. (4). © 2014 The Authors. Published by Elsevier B.V. Open Access under a CC–BY license.

“Normal” energy metabolism of the human brain consists of a complex interaction of multistep processes with trafficking of metabolites between different cells types. In each section of our review (^1^H, ^31^P, and ^13^C), we describe the pathways relevant to the technique, and for review see Ref. (4, 5). It should be noted that normal human brain metabolism remains a subject of research and is still not fully understood, but glucose is invariably considered the principal metabolic fuel for the brain. A simplified schematic of major energy pathways in the brain is shown in Figure 1. After uptake into the brain, most of the glucose is metabolized *via* glycolysis into two molecules of pyruvate, with a net production of two molecules of ATP and two molecules of NADH in the process. A smaller proportion of glucose is metabolized *via* the pentose phosphate pathway (PPP). The PPP is a complex detour starting from glucose-6-phosphate (hence its alternative name “hexose monophosphate shunt”) bypassing some of the steps of glycolysis in the metabolism of glucose. The PPP does not require molecular oxygen, and it does not consume or produce ATP. During the PPP, the first carbon of glucose is lost as CO_2_, NADP^+^ is reduced to NADPH, and various intermediates are produced, including ribose-5-phosphate used in the synthesis of nucleotides and nucleic acids. NADPH participates in reductive reactions such as synthesis of fatty acids and the reduced form of glutathione, a cofactor for glutathione peroxidase. Thus, the PPP has been suggested to play a protective role after TBI, promoting synthesis of molecules for tissue repair and combatting oxidative stress. Therefore, the PPP sacrifices some of the carbon of glucose for the sake of tissue repair. The PPP ultimately rejoins the glycolysis mainstream, and pyruvate may then be incorporated into the tricarboxylic acid (TCA) cycle in cell mitochondria after conversion to acetyl-CoA, where it is metabolized through eight steps, generating three molecules of NADH, one FADH_2_ and a molecule of GTP. FADH_2_ and NADH drive the electron transport chain at the mitochondrial membrane, producing ATP from adenosine diphosphate (ADP) by oxidative phosphorylation in the presence of oxygen. ATP is the fundamental molecule of chemical energy in humans and is used to drive cellular reactions and machinery, being converted back to ADP and inorganic phosphate (Pi) in the process. As an alternative to mitochondrial metabolism, pyruvate may stay in the cytosol and be converted to lactate [by the action of lactate dehydrogenase (LDH)], recycling the NADH produced in glycolysis back to NAD^+^, so allowing glycolysis to continue. The conversion (chemically, an oxidation) of NADH to NAD^+^ in the cytosol can also be accomplished by the action of the electron transport chains of mitochondria, if operational. As NADH cannot itself cross the mitochondrial membrane, the requisite hydrogens and electrons are transferred indirectly by “shuttle” mechanisms. For more information on the above biochemical pathways in the context of the brain, see (6–8).

Studies using a range of techniques have shown that the human brain will take up and directly metabolize alternative fuels such as lactate, acetate, beta-hydroxybutyrate and ketone bodies (4, 5, 9). Shuttling of fuels is also thought to occur between different cell types: the astrocyte-neuron-lactate shuttle hypothesis suggests that astrocytes take up glucose from the blood supply, convert it to lactate, then feed that to their surrounding neurons for conversion back to pyruvate and then metabolism by the TCA cycle (10). A further neuronal–astrocyte coupling is the glutamate (Glu)–glutamine (Gln) cycle, whereby TCA cycle intermediate α-ketoglutarate is converted to Glu for neurotransmission. After Glu is released it is taken up by local astrocytes, converted to Gln, and then fed back to the neurons for conversion back to Glu and thence to α-ketoglutarate, which can reenter (termed anaplerosis) into the TCA cycle, or else Glu can be released for further neurotransmission (5).

Disruption and changes to human brain metabolism following acute severe TBI depend on injury severity and how long after the injury occurred. In the acute phase, a depression of the metabolic rate of glucose and a fall in oxygen consumption is generally reported (11). Brain extracellular lactate may rise following TBI (3, 6), but because lactate is a recognized brain fuel, changes to its absolute concentration are difficult to interpret. More useful is the ratio of lactate/pyruvate as the exchange of these species are at fast equilibrium, directly proportional to the ratio of NADH/NAD^+^ (redox state of the cell) which correlates with outcome following TBI (3, 12).

The metabolic state of the brain and markers of degree of injury can be interrogated with magnetic resonance spectroscopy (MRS), microdialysis, positron emission tomography (PET), and arterio-venous (AV) difference measurements of metabolites. The limitations of microdialysis are its invasive nature involving insertion of intracerebral catheters, its sampling is confined to the extracellular compartment and its highly focal nature means that generalization to the rest of the brain is uncertain. PET is relatively less invasive and reflects the intracellular and extracellular compartments of the brain, but involves the exposure of patients to intravenously injected radioactive (short half-life) ligands, and is usually combined with CT or MRI to enable optimal localization of the PET signal. AV difference studies are invasive and have become less convenient as jugular bulb venous catheters are nowadays not routinely used in the management of patients with acute TBI (2).

Prognosticating in severe TBI can also be difficult. Patient age, neurological status at presentation, and cardiovascular stability are known to correlate statistically with outcome at 6 months (1) but are unable to reliably predict outcome in every individual case. Other biomarkers for prognostication include ICP and the marker of metabolic dysfunction, L/P ratio, which is measured by microdialysis (2). Further prognostic markers that can strengthen existing predictive models of outcome will allow more informed decisions from relatives and clinicians for ceilings of treatment and standardization of injury severity in research studies and clinical audit (1, 2).

*In vivo* MRS allows interrogation of key aspects of brain metabolism and has prognostic value. It is non-invasive, does not involve ionizing radiation, and measures metabolites from whole brain tissue; both the extracellular compartment and also the intracellular compartment [which contributes 80% of total brain volume (13, 14)] of the region selected. Currently, its use is limited to research but this review will discuss the changes in brain metabolites and biomarkers measured by *in vivo* MRS following acute severe TBI, its potential for clinical monitoring to guide treatment, and its value as an additional prognostic tool. A limitation of scan-based technologies such as MRS (also MRI, CT, and PET) is that they give “snapshots” done usually just once or twice during each patient’s neurocritical care, and the question arises of optimally integrating scan-based data with continuous bedside monitoring modalities (2). A detailed description of magnetic resonance (MR) physics is outside the scope of this review and can be found in the literature (15–17). However, we cover a simplified explanation of the relevant basic science of MRS and practical considerations of scanning patients with acute severe TBI.

### Magnetic Resonance Spectroscopy

Certain nuclei possess a property termed “spin” that enables detection by MR. Examples include ^1^H, ^31^P, and ^13^C (which all possess spin of 1/2). Nuclei with 0 spin, e.g., ^12^C, cannot be detected by MR. For illustration, nuclei with spin can be considered as tiny, atomic, bar magnets. MR detection relies on the principle that when a population of magnetic nuclei is placed in an external magnetic field, the nuclei become aligned in a predictable number of orientations. For ^1^H (likewise ^13^C or ^31^P), there are two orientations: with or against the external magnetic field. Since the with-field orientation is preferred as lower energy, slightly more of the population of nuclei are aligned with the field than against the field. Some spins align against the field, as the nuclei are very weak magnets and the energy difference between the two orientations—with and against the external field—is not large, even in a strong external magnetic field. There is enough thermal energy at physiological temperature for nuclei to exchange between the two orientations, though with a slight excess on average in the lower energy (aligned with field) state. MR spectroscopy measurement applies energy as radio-frequency (RF) electromagnetic radiation to excite the small excess of with-field oriented nuclei into the against-field higher energy state. When the RF is removed, the energized nuclei relax back to the lower energy with-field state, and in doing so the relaxing nuclei create their own fluctuating magnetic field. This induces a current in the receiver coil that is around the “sample” (e.g., brain). This current constitutes a signal that is electronically converted into a peak in the spectrum.

For the signal from a nucleus to be detected by *in vivo* MRS the molecule in which it is present must be sufficiently mobile and free to tumble. In the case of nuclei that are bound up in large macromolecules or closely confined by cellular membranes, the spins of the nuclei relax (by spin–spin interaction with other nuclei) too quickly for detection and characterization by *in vivo* MRS.

The RF needed to excite the nucleus depends on what isotope it is (e.g., ^1^H, ^31^P, or ^13^C), its chemical environment and the strength of the external magnetic field, i.e., the scanner magnet (18). The RF needed to excite the nucleus is directly proportional to both the strength of the external magnetic field and the gyromagnetic ratio (see Table 1) of the isotope. The effect of chemical environment is relatively much smaller, but readily measurable. It is due to greater or lesser shielding of the nucleus from the main (external) magnetic field by the electrons surrounding the nucleus. This electron shielding results in small changes of the frequency of the MR signal detected and is called the chemical shift, usually expressed as parts per million (ppm; Hz per MHz). It is the same at all field strengths and is the basis for metabolite identification using MRS. In principle, a peak will be observed for every magnetically distinct nucleus in a molecule because nuclei that are not in identical structural situations do not experience the same shielding, and therefore experience slight differences in external magnetic field.

**Table 1:** Hydrogen, phosphorus, and carbon gyromagnetic ratio, Larmor frequency at 3 T, % natural abundance, and relative sensitivity to ^1^H magnetic resonance spectroscopy accounting for % natural abundance of the isotopes and Larmor frequency, but not natural concentration of biomolecules in the brain.

Isotope	Gyromagnetic ratio (MHz T^-1^)	Larmor frequency at 3 T (MHz)	Natural abundance (%)	Relative sensitivity
^1^H	42.58	127.74	99.99	1
^31^P	17.24	51.72	100.00	0.0665
^13^C	10.71	32.13	1.11	0.00018

*Adapted from de Graaf (15)*.

**Figure 2:**
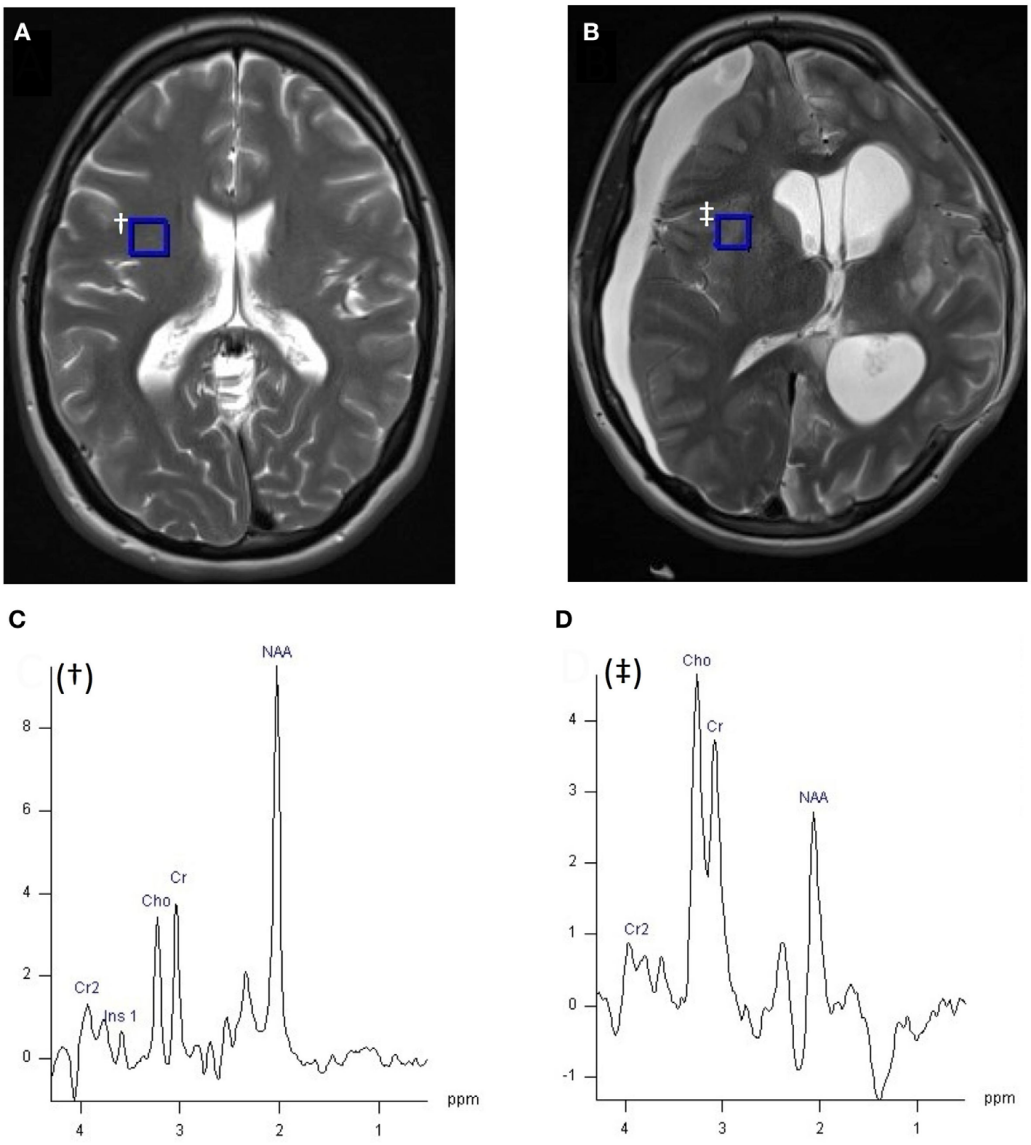
^1^H MRI (T2W axial slice) and ^1^H magnetic resonance spectroscopy chemical shift imaging (CSI) (echo time 135 ms, TR 2,200 ms, 3 averages, 4:41 min acquisition time, 200 ms Hanning filter) of healthy control **(A)**, and patient with acute severe traumatic brain injury after craniectomy **(B)** acquired with a 12 channel ^1^H volume coil on a Siemens 3 T scanner, data analysis performed with Siemens Syngo software. Panel **(A)** demonstrates the position of the selected voxel (blue square, ^†^), represented in panel **(C)**, within the CSI grid (hidden). **(C)** ^1^H spectrum of 10 mm × 10 mm × 15 mm voxel ^†^ from healthy volunteer **(A)**. **(D)** ^1^H spectrum of 8 mm × 8 mm × 15 mm blue square voxel ^‡^ of patient **(B)**, within the CSI grid (hidden). Metabolite peaks are annotated in panels **(C,D)**: Cr, creatine; Cho, choline; NAA, *N*-acetylaspartate, chemical shift on the *x*-axis in parts per million, signal intensity on *y*-axis using arbitrary units. Unpublished images by Tonny V. Veenith, courtesy of the Wolfson Brain Imaging Centre, Cambridge, UK.

**Figure 3:**
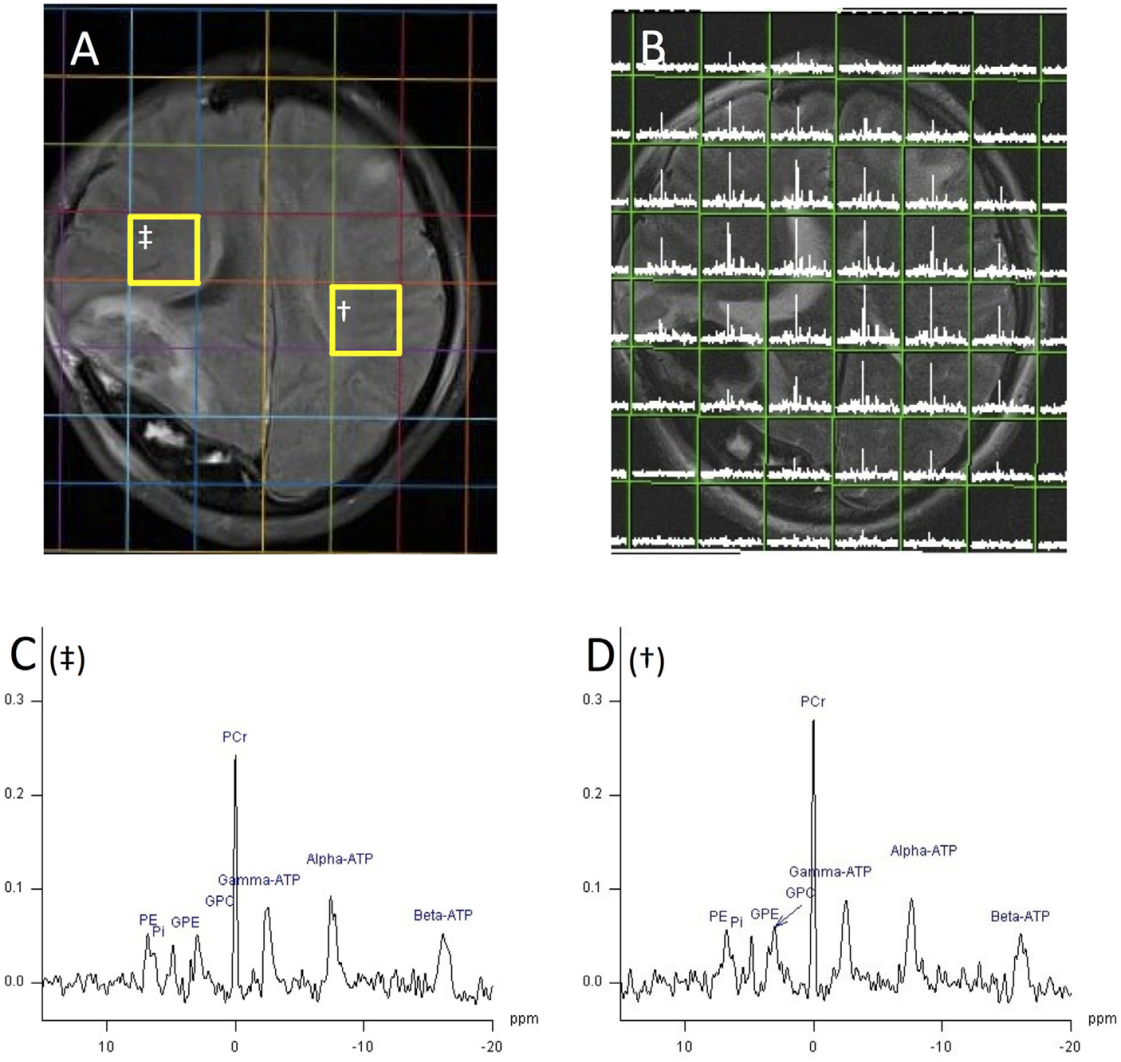
^1^H magnetic resonance spectroscopy (MRI) and ^31^P MRS chemical shift imaging (CSI) (echo time 2.30 ms, TR 4,000 ms, 25 mm voxels, 30 averages, 18 min acquisition time, 200 ms Hanning filter) of patient with acute severe traumatic brain injury acquired with a ^31^P birdcage volume coil (PulseTeq, Chobham, Surrey, UK) on a Siemens 3 T scanner, data analysis performed with Siemens Syngo software. **(A)** Axial FLAIR image demonstrating decompressive craniectomy on patient’s right side with associated regions of high signal in that hemisphere. **(B)** Axial T2 HASTE acquired with ^1^H channel on a ^31^P coil overlaid with ^31^P MRS CSI grid of 8 × 8, 25 mm cubed voxels. Each voxel contains the spectrum from its volume. **(C,D)** ^31^P spectrum from voxel ^†^ **(D)** and ^‡^ **(C)** of image 5A with phosphorus peaks annotated. Species can be identified by their chemical shift on the *x*-axis in parts per million. PE, phosphomonoesters; Pi, inorganic phosphate; GPE, glycerol 3-phosphorylethanolamine; GPC, glycerol 3-phosphorylcholine; PCr, phosphocreatine; ATP, adenosine triphosphate. Signal intensity on *y*-axis using arbitrary units. Unpublished images by the authors, courtesy of the Wolfson Brain Imaging Centre, Cambridge, UK.

Magnetic resonance spectroscopy spectra are typically plotted with chemical shift along the *x*-axis with increasing (positive) chemical shift values reading from right to left (Figures 2 and 3). The *y*-axis represents signal intensity. The size (height and area) and shape of a peak are dependent on the concentration of metabolite(s) that it represents, relaxation time (T1/T2) effects, and splitting by spin–spin coupling. The latter, termed J-coupling, which occurs most strongly between magnetic nuclei that are adjacent to each other causes splitting of their spectral peaks (some splitting by more distant nuclei can also occur). J-coupling can reveal further information about the structure of a nucleus’s molecular environment, but in practice resolution is rarely sufficient with *in vivo* MRS to fully separate a multiplet and so the effect of peak splitting usually just broadens the signal and reduces peak height relative to baseline noise. Spectra can be simplified by ^1^H decoupling, which may be necessary in some applications (see ^13^C MRS), but is of limited value in others (see ^31^P MRS).

**Figure 4:**
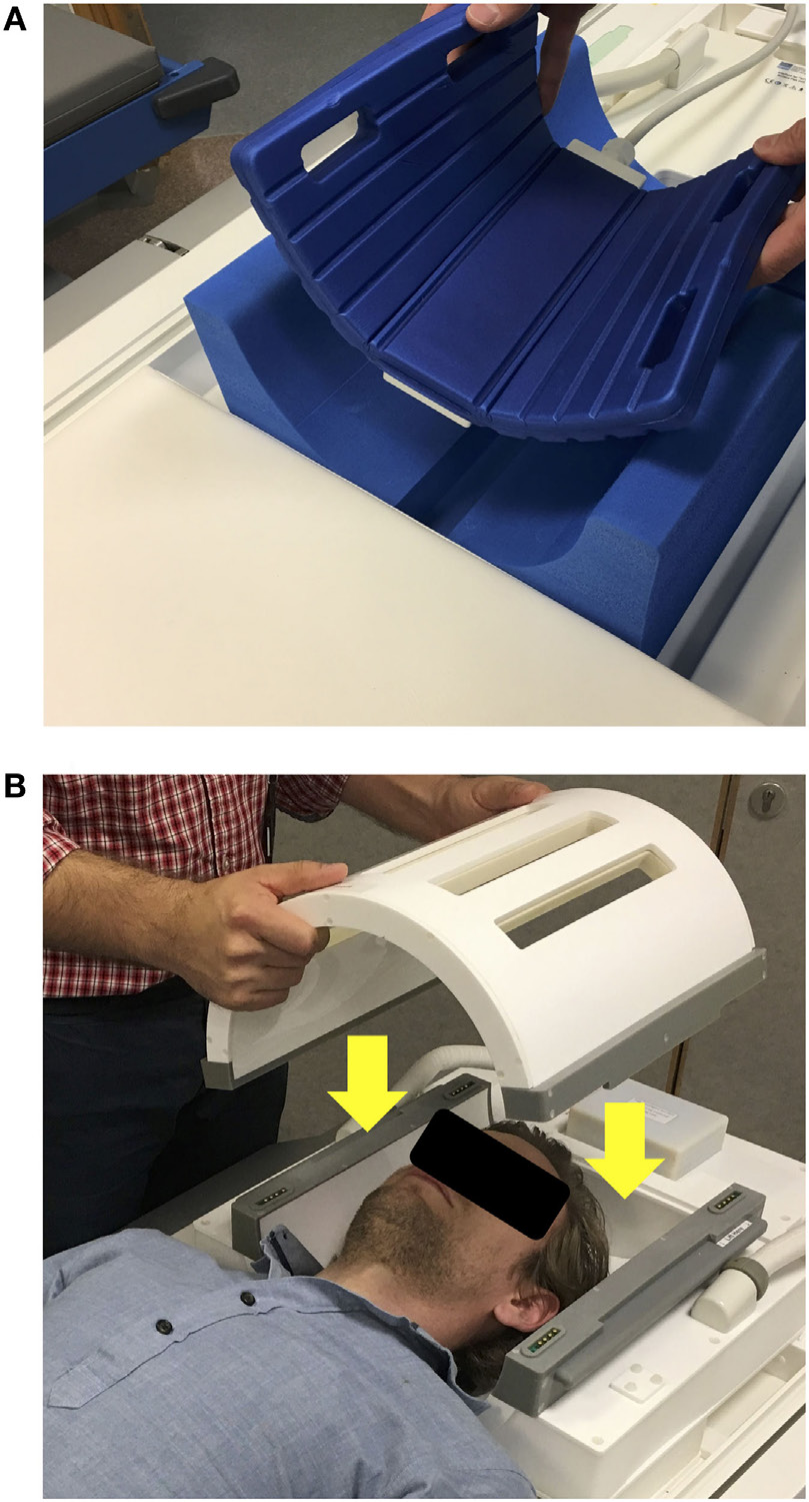
**(A)** Example of a ^13^C surface coil (Rapid Biomedical GmBH, Rimpar, Germany) with flexible design, allowing it to come in closer contact to the patients’ head. Here, it is positioned to sample the occipital lobe. The coil contains a ^13^C channel and ^1^H channel within its housing. **(B)** Example of a ^31^P birdcage volume coil (PulseTeq Ltd., Chobham, Surrey, UK), which can be opened, allowing to access a patient’s head. The coil also contains a ^1^H channel for imaging to allow spectral localization.

As signal frequency differences are used for chemical shift metabolite identification and not for spatial encoding, alternative methods of localization must be used to exclude erroneous signal from non-neural tissue and acquire spectra from chosen regions of interest: a single voxel of brain can be selected using dedicated pulse sequences and gradient magnetic fields such as point-resolved spectroscopy (PRESS), or multivoxel chemical shift imaging (CSI) that uses phase encoding to sample spectra from multiple voxels at the same time (Figures 2 and 3) (18, 19). Outer volume suppression can also be used to suppress signal from scalp and bone (20), and where on a patient’s head a surface coil is placed will affect the region of the brain that it samples. Surface coils (Figure 4A) are more sensitive than volume coils (Figure 4B) that envelope the head, but suffer from a less homogenous delivery of RF pulse to the brain. Due to the different frequencies of ^1^H, ^31^P, and ^13^C, they each require dedicated RF coils that are tuned to their respective frequencies (see Table 1). ^31^P and ^13^C coils will typically contain an additional ^1^H channel within their housing, however, for simple brain imaging to localize the spectra, and for decoupling.

Magnetic resonance scanners are generally classified by their magnetic field strength. Most clinical scanners are either 1.5 or 3 T, which are sufficient for standard MRS studies but higher fields such as 7 or 9.4 T exist. Higher field strength generally results in better spectral resolution and signal-to-noise ratio but comes with the trade-off of greater magnetic field inhomogeneity and RF power deposition into the body resulting in greater tissue heating (21, 22).

*In vivo* MRS studies often express metabolite concentrations as ratios of one another. Whereas the peak area of an MRS spectrum is proportional to the number of excited nuclei within the measurement volume, it is also affected by various other variables: the timing of pulse sequences and their interaction with relaxation times, magnetic field inhomogeneity, and particularly RF coil loading, which will vary between subjects and with coil position (23). To compensate for all these effects is technically very challenging, even with external phantoms, as the latter may not accurately mimic tissue properties, and some biochemicals may be unstable. Expressing metabolites as ratios removes the need for units and calibration although ratios can be more difficult to interpret than absolute concentrations. Application of an artificial reference pseudo-signal is an approach that shows promise for absolute quantification of concentrations in MRS (23).

Quantification of MRS signals, whether absolute or ratios, requires fitting of the spectral peaks. Simple integration to measure peak areas is not adequate in MRS as there is overlap between signals and the spectra are further complicated by noise. Therefore, MRS data are fitted using specialized algorithms that are available as various software packages, e.g., LCModel (24), jMRUI (25, 26), and Syngo on Siemens scanners (Siemens Healthcare GmbH, Erlangen, Germany).

## ^1^H MRS

### Hardware and Sensitivity

As MR imaging of the brain employs the detection of the ^1^H nucleus in water standard clinical head coils can perform ^1^H MRS. With its relatively high sensitivity, this has resulted in ^1^H MRS being the most studied spectroscopy technique in the investigation and monitoring of TBI.

^1^H can be found in most organic molecules, but for a metabolite to be detected by *in vivo* ^1^H MRS it must be present at millimole per liter (mmol/L) concentrations and be freely mobile: not bound to or closely confined by membranes or macromolecules. If it is, the signal from its ^1^H signal decays away too quickly and is either not detected or lost in the baseline (27). As the concentration of water in the brain is ≈50,000 mmol/L ^1^H MRS requires water suppression to stop the huge water peak dominating the spectrum, masking the other metabolites of interest (28).

^1^H MRS is typically performed with a long echo time (TE) of around 120–150 ms (29), which reveals a simplified spectrum of *N*-acetylaspartate (NAA), choline, creatine, and lactate (30). Using a very short TE of around 20–35 ms (28, 31, 32) allows detection of species whose magnetization, and therefore signal, decays more rapidly: Glu, Gln, myoinositol, and lipids. However, the gain in information comes with increased spectral complexity.

### Creatine

The creatine singlet peak at 3.0 ppm in the ^1^H spectrum represents both creatine and its phosphorylated form phosphocreatine (PCr). These are found in high concentrations in metabolically active tissues that require energy in bursts such as brain, muscle, and heart. PCr may rapidly donate its phosphate group to ADP, rapidly regenerating ATP by becoming creatine. In health, creatine is thought to vary less than other ^1^H MRS metabolites throughout the brain so it is the most commonly used denominator when expressing ^1^H metabolite ratios (33). PCr can also be detected by ^31^P MRS (see ^31^P MRS).

#### Effect of TBI

Despite creatine being often regarded as a stable brain metabolite, enzyme extraction studies of rat TBI have shown significant decline (up to 45%) in brain creatine hyperacutely following TBI (34). Conversely, in a human study of mild TBI, creatine was found to be elevated in the splenium of the corpus callosum and white matter of the cingulate gyrus compared to healthy controls, thought to be due to higher energy demand after TBI (35). Many studies do not report a change in creatine after TBI hence creatine is often used as an internal reference for measurement of other metabolites, but these examples demonstrate that the possibility of changes in creatine concentration cannot be ruled out when relying on it as a reference ratio.

### *N*-Acetylaspartate

The NAA peak at 2.0 ppm is a singlet that represents NAA and its product *N*-acetylaspartylglutamate, whose small peak is not resolved from the main NAA peak. NAA is formed from aspartate and acetyl-CoA by l-aspartate *N*-acetyltransferase which are associated with endoplasmic reticulum, or by splitting of *N*-acetylaspartylglutamate by *N*-acetylated-a-linked-amino dipeptidase (27, 29). Its specific role is not fully understood but it is closely associated with mitochondria and ATP (36). NAA is found predominantly in neurons and is thought to be a marker of neuron viability where it is transported down their axons, released, and taken up by oligodendrocytes where it is broken down into acetate and aspartate (35, 37). The role of NAA in myelin lipid synthesis, particularly in early development, is well established. The acetic acid from NAA becomes incorporated into CNS myelin (38). Under metabolic stress, a shortage of acetyl-CoA could result in reduced NAA synthesis and increased hydrolysis of NAA to provide acetate for myelin repair (39, 40). Among other functions ascribed to NAA is the idea that it is involved in osmoregulation (38). Normally, NAA/Cho ratios are higher in gray matter than white matter (29) and can be low due to any cause of neuronal loss. NAA concentrations can be up to 7.5–17 mmol/L in brain; equal to that of the main excitatory neurotransmitter Glu (15, 35).

#### Early Changes after TBI

Studies of hyperacute changes to brain metabolism following TBI are generally limited to experimental animal models due to the time delay transferring patients to hospital and stabilizing them before MRS can be performed. Animal studies show a rapid fall of NAA within the first hours following TBI, proportionally to the severity of the insult that can reach its lowest level at 48 h after injury (41–43). This initial rapid decline in NAA following TBI likely represents a disruption in neuronal NAA production through general micro-architectural disruption and mitochondrial dysfunction (35, 42). Human studies of patients with acute severe TBI performed within 24 h also show a reduction in NAA, NAA/creatine, and NAA/choline compared to healthy controls (44–47). Another study of 10 patients with moderate–severe TBI studied slightly later, after 48–72 h after injury also found a reduction in NAA in ^1^H MRS compared to healthy volunteers, and the reduction was correlated with injury severity (GCS at presentation) (48).

^1^H MRS performed in the subacute period around 1 week following acute severe TBI typically demonstrate persisting lower NAA/creatine ratios than healthy controls (29, 49, 50), which continued to fall in one study (29). Interrogation of peri-lesional brain typically showed even greater NAA decline through the subacute period, beyond 10 days (29).

#### Later Changes after TBI

If the primary injury is not too severe or compounded by further metabolic stress such as hypoxia or hypoperfusion, mitochondrial function and NAA may recover over the preceding days and months (41) with preservation of the neuron population. If the injury is more severe, there is likely irreversible physical and metabolic damage to the neurons which leads to a significant decline in neuronal population and therefore no recovery of NAA on MRS studies.

Studies of delayed ^1^H MRS performed in the chronic, recovery phase after acute TBI either show recovery of NAA back to the levels seen in healthy controls in patients who make a good recovery or a persisting depression of NAA measured by ^1^H MRS in patients with poor long-term neurological outcome (29, 51). An exception to this is regions of brain surrounding significant traumatic lesions which tend not to recover despite patients having a good recovery (29), and a study by Garnett et al. who found persisting NAA depression in all patients, regardless of outcome (49). Contrastingly in other pathologies, partial recovery of brain NAA levels was reported using ^1^H MRS in a small follow-up study of acute brain damage (non-TBI) patients (52).

Chronic NAA depression may affect white matter more than gray matter following severe TBI, as studies of patients at 6 weeks to 6 months after TBI found reduced NAA in the white matter and not gray matter (51, 53). This may also be explained by most studies selecting regions of the brain predominantly represent white matter and the corpus callosum.

#### Role in Clinical Care

Measuring NAA using ^1^H MRS can be clinically valuable due to its correlation with patient prognosis: the severity of depression of NAA/total metabolites (48), NAA/Cho (29), and NAA/Cr (49, 54) measured in the acute and subacute phase of injury correlates with patient outcome. Whereas these studies predominantly selected subcortical white matter and corpus callosum, the recovery of NAA in the thalami of TBI patients acutely after injury has been shown to predict good outcome (55). Another study of brainstem ^1^H MRS in 40 patients with severe TBI showed that at a median 17 days after injury NAA/Cr ratio could predict very poor outcome in some patients that did not have visible injury on MRI. Furthermore, when included in a principal component analysis with FLAIR and T2* imaging, MRS allowed accurate prediction of GOS I–II, GOS III, and GOS IV–V outcomes when these modalities alone could not (56).

### Choline

The choline peak at 3.2 ppm is formed from free choline, phosphocholine, and glycerophosphocholine (15). Choline is a precursor of acetylcholine; an important neurotransmitter that is also found at high concentrations bound to cell membrane phospholipids. In its bound form its T2 is too short for detection, but when it is liberated during cell membrane turnover or cellular production of acetylcholine it becomes visible. An increase in choline is used to identify increases in cell membrane turnover or destruction in aggressive brain tumors and demyelinating disease, but in normal brain it is found at 0.5–2.5 mmol/L (15).

#### Early Changes after TBI

Following TBI, a raised choline is thought to represent cellular damage through membrane breakdown. Elevated choline/creatine compared to healthy controls has been found both subacutely after injury and in the chronic phase (49, 57). Garnett et al. found choline/creatine increased in proportion to the severity of injury in normal-appearing white matter (49), but Wild et al. found no such correlation (57), although this could be due to changes in creatine blunting the effect of any relative change. An elevation of choline/total metabolites has been demonstrated within 48–72 h of moderate–severe TBI, but this also did not correlate with presentation GCS or outcome at 3 months (48).

#### Later Changes after TBI

^1^H MRS performed in the subacute period following moderate-minor TBI of 40 patients found elevated choline/NAA ratio throughout the cerebrum and cerebellum (56). However, there was an inverse relationship with outcome as patients with higher choline/NAA ratios had better cognitive performance at recovery. Delayed choline measurement during the chronic phase of severe TBI recovery often demonstrate persisting elevated choline/creatine and reduced NAA/choline (49, 51) that sometimes correlates with functional status at the time (58).

#### Role in Clinical Care

Choline can potentially be used as a predictor for TBI prognosis. A study of 42 patients with subacute (7 days post-injury) severe TBI found that choline elevation in occipital gray and parietal white matter predicted outcome with 94% accuracy (32). However, a separate smaller study (10 patients) performed in the acute period (48–72 h) did not find a correlation with degree of choline elevation and outcome (48). It is not clear why the magnitude of the acute choline rise does not correlate with the severity of the initial injury or later functional outcome of the patient. Delayed choline measurements tend to be more closely associated with outcome (32, 49) which may be because choline represents active neuroinflammation causing further cell membrane disruption and injury, well after the initial TBI (59, 60). If this is the case, ^1^H MRS could be used to identify patients at risk of neuroinflammation; selecting them for potential new anti-neuroinflammatory therapeutic agents (61).

### Myoinositol

Myoinositol is a precursor of both phosphatidylinositol and phosphatidylinositol 4,5-bisphosphate. Its ^1^H MRS peak is at 3.56 ppm and normal concentration in the brain is 4.0–9.0 mmol/L. It is regarded as a cerebral osmolyte and astrocyte marker. Variable changes are seen in different intracranial pathologies: an absolute decrease may be seen in stroke and hepatic encephalopathy, likely due to imbalance of osmoregulation, while an increase in myoinositol is found in astrogliosis, although when this is expressed as a ratio of myoinositol/creatine the effect disappears (62).

#### Effect of TBI

Pascual et al. showed that myoinositol can increase in the first 24–48 h after TBI in a rat model (63). A study of 38 pediatric TBI patients showed occipital gray matter myoinositol levels were increased in children with TBI compared to healthy controls and that higher myoinositol levels correlated with poor outcome (64).

### Glu and Gln

Glutamate and Gln are amino acids found in abundance in the human brain detected at 2.2–2.4 ppm in a ^1^H MRS spectrum. Glu is the main excitatory neurotransmitter in the brain and is stored in neuron vesicles, found at a concentration between 6.0 and 12.5 mmol/L in healthy human brain (15). After release, it is taken up by glia and converted to Gln, which is then fed back to neurons in the Glu–Gln cycle (65). Gln is found in the brain at concentrations of 3.0–6.0 mmol/L (15). The molecular structure of Glu and Gln is sufficiently similar that it is difficult to distinguish between their chemical shifts [2.04–2.35 and 2.12–2.46 ppm (15)] on an *in vivo* ^1^H MRS examination. Thus, the term “Glx” is used to represent the combined pool of both metabolites.

#### Effect of TBI

During TBI, there may be intensive neuronal activation associated with impaired Glu reuptake and transport that causes Glu associated excitotoxicity (32, 66, 67). Shutter et al. found combined Glu and glutamine (Glx) were significantly elevated in occipital gray and parietal white matter early after injury (7 days) in patients with poor outcome at 6 and 12 months after TBI and combined Glx and Cho ratios predicted long-term outcome with 94% accuracy when GCS motor score was included in the model (32).

### Gamma-Aminobutyric Acid (GABA)

Gamma-aminobutyric acid is the main inhibitory neurotransmitter of the brain and, like Glu, is stored intracellularly in neuron vesicles at concentrations of up to 1 mmol/L in the brain (68). After release, it is taken up by glia and converted to Gln *via* Glu and fed back to neurons. Its ^1^H MRS peak is found between 2.2 and 2.4 ppm which overlaps with the Glx species and thus is very difficult to quantify (68, 69). GABA plays a role in epilepsy and can be increased in patients with epilepsy by treatment with common anticonvulsants. However, other studies have shown no difference between patients suffering with epilepsy and normal healthy controls (68). GABA quantification can be improved by acquiring the spectra using specialized GABA-editing techniques such as the pulse sequence MEGA-PRESS (70, 71).

#### Effect of TBI

Gamma-aminobutyric acid normally modulates the excitatory pathways in the brain. Following TBI a loss of GABAergic neurons disrupts the balance of excitation and inhibition, leading to further cell injury and apoptosis (72). An imbalance of GABA and Glu after TBI may also result in post-traumatic epilepsy but measurements of GABA are rarely reported in human ^1^H MRS studies and GABA has only been shown to fall after TBI by 46% within 24 h in a single animal study.

### Lactate

Most of the lactate in the brain is regarded as “glycolytic,” originating from glucose metabolism *via* the Embden–Meyerhof pathway, to pyruvate, followed by conversion of pyruvate to lactate by the action of LDH. There is some disparity in nomenclature about glycolysis in the brain literature, which undoubtedly adds confusion, as glycolysis culminating in lactate is often termed “anaerobic metabolism,” though often without supporting evidence regarding the oxygen status in the tissue concerned. In old studies brain injury was often associated with hypoxia/ischemia (real or assumed), although modern neurocritical care means that overt hypoxia/ischemia is usually avoided. Even so, microvascular ischemia appears to exist in some cases (73), as do episodes of hypoxia (74). We regard hypoxia as PbtO_2_ <20 mmHg, with severe hypoxia as PbtO_2_ <10 mmHg.

The ability of lactate to act as a neuronal fuel has now also been established (6, 75) although its importance relative to glucose is debated (76). Lactate may be elevated by hypoxia, ischemia, or macrophage infiltration (77). It can appear as a characteristic doublet at 1.3 ppm when acquired with a long TE (TE 144 ms), but the MR behavior of lactate is complex, and lactate signals can virtually disappear or appear inverted depending on MR conditions (78). Interpretation of lactate is further complicated by overlap with lipid signals. Lactate is typically represented by a small peak on ^1^H MRS despite its relatively high extracellular concentration of 2.9 mmol/L (79) as its concentration intracellularly [which dominates brain volume (13, 14)] is much lower.

#### Effect of TBI

In TBI, the elevation of brain extracellular lactate is known to be associated with poor prognosis. Although lactate is a normal component of energy metabolism, if lactate appears elevated in a tissue on ^1^H MRS it is usually a sign of pathology. Lactate elevation does not necessarily indicate hypoxia, as the phenomenon of “aerobic glycolysis” whereby cells produce lactate despite a seemingly adequate supply of oxygen is well known, e.g., the Warburg effect in tumors, and a similar effect is seen in TBI, where it is variously termed metabolic dysfunction, mitochondrial dysfunction, and, in extreme cases, metabolic crisis. In early work on rat models of TBI there appeared to be an initial rise in brain lactate hyperacutely following moderate or severe injury, associated with persisting neurological dysfunction at 4 weeks (80, 81). Lactate returned to normal after about 60 min, and there was no association between magnitude of hyperacute transient lactate rise, injury severity or neurological outcome. However, mild injury that did not result in long-term neurological deficit did not cause any increase in lactate (80). The hyperacute period is only addressable in experimental models, and study is not feasible in human TBI patients, as typically an hour or more will have elapsed before they arrive at hospital and longer until a scan can be performed. In human TBI, lactate elevation can be seen on ^1^H MRS in some but not all instances, illustrated in Marino et al. (48). Because of the complications with lactate signals (see above) some ^1^H MRS studies of normal and TBI brain do not consider lactate at all (82). Lactate elevation is most markedly seen in pediatric head injury (83, 84). Makoroff et al. showed that in four pediatric TBI patients elevation of lactate measured by MRS was due to hypoxic–ischemic injury, which was associated with worse early neurological outcome scores (85). In adult TBI patients, lactate (measured by MRS) is similarly only raised if there is a severe ischemic process where it may rise diffusely in the brain within 48–72 h of injury (48). This rise can persist for weeks (86), and the degree of lactate elevation may correlate with outcome at 3 months; higher lactate corresponding to worse outcome (48).

### Lipid

Lipids and phospholipids form a group of peaks at 1.3 ppm. When lipid is bound in intact cell membranes its T2 is too short for detection by *in vivo*^1^ H MRS. Elevated lipid suggests significant cell membrane disruption so is only visible in severe trauma, such as in shaken baby syndrome (87). Lipid measurements are not often reported in adult TBI studies.

### Summary of ^1^H MRS in TBI

Following TBI, the brain may suffer from significant metabolic failure, direct cell damage, hypoxia, and neuroinflammation. These can be detected non-invasively using ^1^H MRS, prompting intervention: metabolic failure signified by NAA reduction may allow a patient’s metabolic support to be altered by administering an infusion of glucose, or newly developing metabolic treatments for mitochondrial failure such as succinate (88).

Prognosticating in acute severe TBI is challenging. Several metabolites, including NAA, choline, myoinositol, Glx, lactate, and lipid may help predict patients who will not survive or are likely to survive with the most extreme disability (32, 48, 49, 54–56). ^1^H MRS can help clinicians and patients’ families in terms of prognosis. As acute severe TBI typically results in both a fall in NAA and a rise in Cho that are associated with outcome, the NAA/Cho may be the most appropriate indicator of injury, distinguishing patients with good and poor outcome (32). This has the potential to reduce patient and family suffering and conserve intensive care resources.

The most appropriate region of the brain to be interrogated for prognostication is unclear. CSI measurements of the subcortical white matter with inclusion of the corpus callosum would be the most comparable to the literature (29, 48, 49, 54), and the inclusion of single voxel brainstem NAA measurement would allow MRI invisible injury to this critical structure to be detected (56). Other potential targets are the occipital and parietal lobes where changes in Glx, myoinositol, and Cho have been correlated with patient outcome.

A summary of the effect of TBI on metabolites interrogated by ^1^H MRS are shown in Table 2.

**Table 2:** Summary of metabolite changes following traumatic brain injury (TBI) detectable with *in vivo* ^1^H magnetic resonance spectroscopy.

	Spectrum peak (ppm)	Physiology	Change in acute TBI	Change in chronic TBI	Correlation to prognosis
*N*-acetylaspartate	2.02	Neuron viability	↓↓	↓	✓
Creatine	3.02	Adenosine triphosphate generation and energy metabolism	↔		
Choline	3.24	Cell membrane turnover	↑↑	↑	✓
Myoinositol	3.5	Osmoregulation	↑		✓
Glx [glutamate (Glu) + glutamine]	2.2–2.4	Excitatory neurotransmitter (Glu)	↑↑		✓
Lactate	1.33	Mitochondrial dysfunction	↑		✓
Gamma-aminobutyric acid	2.2–2.4	Inhibitory neurotransmitter	↓		
Lipid	1.3	Cell membrane	↑		✓

*↑: increase in metabolite; ↓: decrease in metabolite; ↔: no significant change or insufficient data; ✓: potential clinical use as a prognostic predictor*.

## ^31^P MRS

*In vivo* ^31^P MRS detects unbound molecules that contain phosphorus in the human brain. The most notable of these are the fundamental molecules of chemical energy in all eukaryotic organisms: ATP, ADP, adenosine monophosphate (AMP), PCr, and Pi. As well as providing information about energy status, Pi allows measurement of brain pH through changes in its chemical shift (89–91). Phosphomonoesters (PMEs) and phosphodiesters (PDEs) are also metabolites that contribute to a standard ^31^P brain spectrum and are thought to represent cell membrane turnover.

### Hardware and Resolution

Magnetic resonance spectroscopy detection of ^31^P is less sensitive than ^1^H. Comparing the two isotopes, for the same number of nuclei in the same external magnetic field, the relative sensitivity, also termed receptivity, is calculated from the NMR sensitivity [proportional to |γ^3^| × *I* (*I* + 1)] multiplied by the natural abundance (15). Since *I* (the spin) is 1/2 for both ^31^P and ^1^H, and the natural abundance is over 99.9% for ^1^H and 100% for ^31^P, the gyromagnetic ratio γ is the crucial factor: 26.752 and 10.831 (units 10^7^ rad T^−1^ s^−1^) so relative sensitivity (vs. ^1^H) is only 0.065 for ^31^P, so just 6.5% (15). In layman’s terms, the gyromagnetic ratio γ, can be thought of as the strength of the tiny magnets that are the ^31^P and ^1^H nuclei, divided by their spin (value 1/2 here in both cases)—thus ^31^P is less sensitively detected than ^1^H, because the ^31^P nuclei are weaker magnets than ^1^H nuclei.

To acquire phosphorus spectra with acceptable signal to noise, either larger voxels must be selected compared with ^1^H MRS and/or more averages acquired, resulting in longer scan times. ^31^P MRS is also limited by the pulse sequences for localization that can be used: ^31^P metabolites have relatively short relaxation times so the transverse magnetization must be sampled as quickly as possible after excitation (short TE). Single volume spectroscopy techniques PRESS and STEAM use multiple echo steps that require long TE, so ^31^P MRS localization is limited to single voxel ISIS and multivoxel CSI in the brain (92).

The range of chemical shifts that the main metabolites in an *in vivo* ^31^P MRS spectra occupy is also much wider (≈30 ppm) than that of ^1^H MRS (≈5 ppm). The chemical shifts of PCr and Pi are dependent on pH, and α-ATP and β-ATP on the concentration of free magnesium (Mg^2+^). PCr is conventionally considered a reference at 0 ppm (by definition), and the chemical shifts quoted below represent those from its center at a pH of 7.2 with normal tissue Mg^2+^, as per de Graaf (15).

### High-Energy Phosphates

The high-energy phosphates detected by ^31^P MRS (PCr, ATP, ADP, AMP, and Pi) are directly related to each other chemically: the high-energy phosphate group passes from pool to pool reaching a state of equilibrium depending on the energy expenditure and generation within the cells. This contrasts with metabolites studied by ^1^H MRS which are linked to each other in a broader, biological sense. Thus, we will consider the high-energy phosphates as a group, with relative ratios of interconnected metabolites; rather than as individual species.

#### ATP Hydrolysis and Generation

Adenosine triphosphate is the fundamental molecule of chemical energy in eukaryotic and prokaryotic organisms and is used and then regenerated with rapid turnover in the brain (93). The hydrolysis of ATP into ADP + Pi releases energy that is harnessed to drive the main cellular processes including the sodium potassium exchanger (Na^+^/K^+^ ATP_ase_ pump) that maintains the membrane potential in neurons. The brain maintains ATP at a concentration several fold higher than that of ADP [average 3 vs. 0.1 mmol/L (15, 94, 95)] to drive these processes by continually recycling ADP back to ATP. This is done through glycolysis, the citric acid cycle and the electron transport chain in mitochondria where the enzyme ATP synthase catalyzes the conversion of ADP and Pi to ATP down a hydrogen ion gradient, provided oxygen is available as a terminal electron acceptor on complex IV. This cycle occurs continually so that the human brain, weighing about 1.2 kg, uses an estimated 5.7 kg of ATP per day (93).

#### Creatine Kinase

The process of ATP regeneration *via* ATP synthase is relatively slow on a cellular scale so tissues that require energy in bursts such as the brain, skeletal muscle, and cardiac muscle contain creatine and PCr. Catalyzed by the enzyme creatine kinase, PCr can very rapidly donate its high-energy phosphate group to ADP, rapidly regenerating ATP during periods of high metabolic demand independently of oxygen. During periods of lower metabolic demand, the PCr store is replenished in the mitochondrial intermembrane space, again by creatine kinase from newly generated ATP. PCr is a spatial buffer for ATP as well as a temporal buffer. Most ATP is produced in the mitochondria but used in the cytoplasm. The free diffusion distance of ATP and ADP is limited by their strong negative charges and low cellular concentrations whereas PCr and Cr diffuse more freely due to their smaller size, less overall charge, and higher concentrations. The PCr–Cr system therefore acts as a shuttle linking ATP production in the mitochondria to its use in the cytoplasm (96–99).

### ^31^P Peaks and Their Metabolites

The PCr signal, whose chemical shift is defined by convention as 0.00 ppm, is the most easily identifiable peak in brain ^31^P MRS. Brain PCr concentration has been reported at 4.0–5.5 mmol/L (15) concentrations at reasonably constant levels between gray and white matter (15, 100, 101).

The β-ATP peak represents phosphorus in the middle phosphate group; a structure that is unique to ATP (15). It would appear to be the most appropriate peak to represent ATP concentration, but its location at extreme upfield (−16.26 ppm) can make it difficult to excite consistently with a homogenous RF pulse that also covers the other metabolites.

γ-ATP is often used to represent the concentration of ATP, instead of β-ATP. At −2.48 ppm γ-ATP represents the distal phosphate groups of both ATP and ADP, which are effectively indistinguishable from each other *in vivo* due to their similar immediate chemical and nuclear environment. However, ADP is found at much lower concentrations in the brain (0.1 mmol/L) than ATP (3 mmol/L) (15, 94, 101), and ADP is regarded as mostly bound up in vesicles and mitochondria so poorly responsive on MR, making its contribution to the γ-ATP peak negligible.

α-ATP at −7.52 ppm represents the proximal phosphate groups in ATP and ADP and the central phosphates of NAD and NADH; these are poorly resolved in most *in vivo* MR spectra. The inclusion of NAD and NADH and its profile slightly further from the center of a typical RF pulse makes it an inferior choice to the γ-ATP peak for ATP characterization (15).

Inorganic phosphate is found at 5.02 ppm as a relatively small peak. Its small size can make it difficult to accurately integrate, but nevertheless it is often used to express ratios of brain energy ^31^P species (102–104). Pi is a useful indicator of intracellular pH, which can be calculated from the difference in chemical shift between PCr and Pi (90, 105). Although Pi is may be a small peak some studies have shown existence of two Pi signals; ascribed to two pools of Pi differing in pH (ΔpH ~0.4) (106). In brain, the major (upfield) peak is assigned as intracellular Pi, and the minor (downfield) peak extracellular Pi, and the two signals have different T1 relaxation times, presumably reflecting the different environments surrounding the phosphate molecules.

#### Changes after TBI

PCr/ATP and PCr/Pi are two of the most commonly used ratios to express brain energy status. If the brain is metabolizing normally there will be sufficient ATP and plenty of its short-term high-energy store, PCr. However, if the brain is stressed, a plausible scenario is that it might draw on its store of PCr to maintain ATP homeostasis leading to a reduction in the PCr/ATP ratio and PCr/Pi ratio. The PCr/Pi ratio will also be affected by a potential increase in free Pi as ATP is hydrolyzed but not remade sufficiently in the mitochondria. PCr/Pi can be inaccurate with difficulty in reliably measuring the small Pi peak in a potentially noisy baseline.

Hyperacute ^31^P MRS studies of TBI are limited to animals for the same reason as ^1^H MRS. Ishige et al.’s study (103) of focal TBI in rats with sequential measurements after injury demonstrated a rapid fall in absolute PCr and an increase in absolute Pi in the first 15 min after injury. In the absence of further injury, these species recovered to near normal within 90 min (103). Further studies by Vink et al. of different grades of injury have demonstrated the same initial fall in absolute PCr and rise in absolute Pi (or fall in PCr/Pi ratio), which then recovers within ~100 min following moderate–severe trauma. There appears to be a second rise in PCr and fall in Pi and PCr/Pi ratio that occurs 120 min after injury, remaining depressed in severe injury (104, 107, 108). The initial falls in PCr/Pi were associated with brain acidosis in these studies, but the second falls were not. The degree of this second PCr/Pi depression 4 h after injury correlated with severity of insult, which itself correlated with 24 h neurological dysfunction (107). No studies demonstrated a decrease in ATP after moderate–severe injury. In the studies that included the most extreme injury severity, a different pattern was observed: a much greater, persistent fall in PCr and rise in Pi occurred that did not recover (108). Unlike animals subjected to more moderate grades of injury these animals also experienced an irreversible loss of ATP over the 3 h following injury (107, 108).

The addition of a secondary insult, hypotension, to experimental TBI greatly exacerbated the metabolic derangement measured by ^31^P MRS. With moderate hypotension after TBI, a much greater immediate fall was seen in PCr which did not recover as well. Pi increased significantly more and the immediate acidosis was greater and did not recover as it did in the absence of hypotension. Importantly, ATP fell significantly in the presence of moderate–severe TBI with hypotension but not with TBI alone. Cells work very hard to maintain ATP homeostasis at the cost of other metabolites, so it appears that a fall only occurs in metabolic extremis following very severe injury, or when TBI occurs with additional hypotensive insult (103, 107–109).

An *in vivo* ^31^P MRS patient study by Garnett et al. (102) of high-energy phosphates in the subacute period following TBI had different findings to those of the hyperacute TBI animal studies above. Seven patients with moderate and severe TBI were studied 9 days (mean) after injury: four patients had partially recovered and were self-ventilating whereas three were still intubated and ventilated. In normal-appearing white matter, a significant increase in PCr/Pi was found in patients with TBI compared to healthy controls, as was PCr/ATP (although non-significantly). The authors suggested that a possible explanation could be a change in cell population through reactive gliosis.

These studies suggest that ^31^P MRS is detecting different changes in brain metabolism dependent on when after the injury the scan is performed. The initial fall in PCr seen in hyperacute animal studies (see above) is compatible with the interpretation of cell membrane injury causing K^+^ efflux from cells, which leads to high demand on the Na^+^/K^+^ ATPase pump, consuming PCr. This initial fall in PCr recovered in these animal studies, but the second fall in the acute stage after 2 h did not and is of uncertain etiology. Cellular ATP appears to be maintained following all but the most severe forms of experimental TBI in animals, likely representing catastrophic energy failure with extreme, irreversible derangement of all phosphorus metabolites (103, 107, 108).

### Brain pH and Mg^2+^ Concentration

The pH of the brain can be measured from the difference in chemical shift between the Pi and PCr peaks (89–91). Although the small size of the Pi peak relative to baseline noise can lead to errors measuring its area, its chemical shift can generally be accurately identified. Changes in the concentration of hydrogen ions (pH) result in greater or lesser binding of H^+^ ions to Pi. The presence of the additional hydrogen ions changes the proportion of protonated to un-protonated Pi which changes the mean chemical shift of the species population. Similarly, the concentration of brain Mg^2+^ can be calculated from the difference in chemical shifts between the α-ATP and β-ATP peaks (15, 89, 103, 104, 110).

#### Control of Brain pH

Normal neuronal activity causes constant changes in intracellular and extracellular pH in the brain which are buffered by several mechanisms: the PCr, ATP, and creatine kinase system is one of these. When creatine kinase catalyzes the regeneration of ATP from ADP and PCr, a H^+^ ion is consumed: ADP + PCr + H^+^⇆ ATP + Cr. Creatine kinase is strongly pH dependent and acts as both an ATP and pH buffer in cells with high metabolic workloads.

#### Effect of TBI

Rodent studies of hyperacute changes in brain pH following severe TBI have found an immediate, transient fall in pH for the first 15–60 min following moderate to severe TBI that is exacerbated by hypotension (80, 103, 104, 111). The magnitude of this transient acidosis does not correlate with neurological outcome, histopathological injury or severity of insult (80) for all but the most extreme (un-survivable) injuries where a progressive, terminal brain acidosis occurs (108). Changes in pH accompany changes in PCr/Pi ratio, returning to normal after an hour and a half in the absence of hypotension. This is what would be expected from the creatine kinase system, but it is not clear if a fall in PCr causes a shift of the equilibrium, and a rise in H^+^ ions, or acidification causes a shift in the CK equilibrium and a fall in PCr. It is perhaps more likely that primary pH changes drive the PCr/Pi change as the delayed fall in PCr/Pi does not cause a change in pH, suggesting another mechanism.

Intracellular free Mg^2+^, an important cofactor for glycolysis and oxidative phosphorylation, has been shown to fall by as much as 60–69% following animal experimental TBI (110–112), reaching its nadir between 1 and 4 h after injury. Free Mg^2+^ appears to be particularly sensitive to injury; declining significantly following moderate and even mild experimental TBI in the absence of changes to PCr, ATP, Pi and pH detected by ^31^P MRS (43, 108, 110–112). Interestingly, in a graded TBI study performed by Vink et al. free intracellular Mg^2+^ did not fall in rats subjected to the most severe TBI. This was attributed to release of Mg^2+^ from the declining ATP that occurred in this group, replenishing the total level. After moderate injury Mg^2+^ appears to recover to baseline after about a week (43), but its calculation should be performed cautiously when spectra have low signal to noise as previous reported changes have been shown to be due to errors of chemical shift assignments (89). The subacute study by Garnett et al. of patients with moderate to severe TBI found white matter was more alkaline (higher intracellular pH) and had higher free intracellular Mg^2+^ in TBI patients 2–21 days (mean 9 days) after injury compared to healthy volunteers (102). A difference in gray matter pH was not found, although gray matter PCr/ATP was significantly higher in TBI brain than in healthy controls (102). Conversely, measurements of brain *extracellular* pH (not using MRS, but using intracranial probes) following severe TBI in humans suggest that lower pH is associated with a worse outcome (113, 114). The relationship between brain extracellular and intracellular pH in human TBI is unclear.

### PMEs and PDEs

The cell membrane phospholipid bilayer in the brain is not visible on ^31^P MRS because its magnetization decays too quickly for detection. Its precursors the PMEs phosphorylethanolamine (PE) and phosphorylcholine, are visible at 6.78 and 5.88 ppm in high quality spectra (15, 102). PDEs glycerol 3-phosphorylethanolamine and glycerol 3-phosphorylcholine, at 3.2 and 2.8 ppm, are produced by phospholipase breakdown of cell membranes. They are then converted to PMEs by phosphodiesterase. Consequently, the ratio of PME/PDE is thought to be an indicator of cell membrane turnover (92, 95, 115).

Changes in PME/PDE ratios are often explored in ^31^P MRS TBI studies, but the small size of the PME and PDE peaks relative to baseline noise means that statistically significant differences often cannot be found, even if present (102). However, it should be noted that the phosphorus nuclei in PMEs and PDEs are coupled to hydrogen atoms, which causes splitting of their resonances that can be exploited with the polarization transfer technique and proton decoupling to significantly enhance their detection (15).

### Confounders of ^31^P MRS Measurements in the Brain

Regional variations of high-energy phosphate species in the human brain exist that influence the results obtained by ^31^P MRS studies. Whereas the concentration of PCr remains relatively constant throughout the brain, PCr/ATP is higher in gray matter (GM = 1.19) than white matter (WM = 0.84) (116) because of the higher concentration of ATP found in white matter (GM = 2 mmol/L; WM = 3.5 mmol/L) (15). GM also has a higher metabolic rate than WM, using three times as much ATP and consuming 77% of total energy expenditure of the brain despite representing only 55% by tissue weight (116).

Phosphocreatine is known to vary with age in healthy volunteers: increasing age is associated with slightly higher PCr, lower PME and a slightly more acidic brain (117). There is also an inverse relationship between body mass index and absolute measures of PCr and ATP, but as these changes are equivalent there is no resulting change in PCr/ATP ratio (118).

If patients with acute severe TBI are studied whilst intubated, sedated and ventilated the effect of anesthetic agents should also be considered. There is evidence that phenobarbital increases the PCr of rat brain but does not change ATP or ADP, measured by biochemical assays on tissue extracts (119). Halothane, nitrous oxide and fentanyl do not seem to have any effect on high-energy phosphates concentrations (119).

### Magnetization Transfer (MT) Technique

As well as measuring static concentrations of phosphorus metabolites (absolute and ratios), flux from one pool to another can be measured using the MT technique. MT is technically challenging compared to “standard” ^31^P MRS. The basis of MT is selective saturation or inversion of a resonance of one moiety which undergoes chemical exchange to another. If the rate of exchange is fast compared to T1, then the saturation or inversion is transferred; quantification of exchange rates requires knowledge of the T1 and MT rate (120). MT can provide information on the flux between PCr and ATP and hence the rate of creatine kinase (121, 122). Similar methodology has also been applied to assess the flux between Pi and ATP to estimate ATP synthesis rate in brain (106, 145). However, concern surrounds this technique as ATP synthesis rates from MT transfer are significantly higher than the rates of oxidative ATP synthesis measured by other techniques, shown in muscle, heart, and liver (123, 124). This discrepancy is usually attributed to rapid Pi-ATP exchange *via* glycolysis, which can produce significantly higher MT measures of Pi → ATP flux compared with net oxidative Pi → ATP flux (123–126). Although this does not necessarily invalidate MT measures of ATP synthesis rates in brain (145, 127), where average measures agree with rates calculated indirectly from previously reported cerebral metabolic rate of glucose consumption (145), varying levels of anesthesia in TBI may also influence results.

### Therapeutic and Prognostic Potential

^31^P MRS studies have shown changes in PCr, Pi, pH, and Mg^2+^ in the brain following TBI, with ATP being relatively unaffected except for under extreme stress in experimental TBI, although timing of when after injury a ^31^P MRS study is performed is key.

In a clinical setting, ^31^P MRS cannot be performed in the hyperacute period after injury because of the time required transferring a patient to hospital and stabilizing them. However, if a patient displayed severely depressed PCr/Pi and pH measured by ^31^P MRS on the day of injury, this may suggest that the initial injury was extreme, or compounded by a period of hypotension, which may or may not have been known about. As well as prompting meticulous control of cerebral perfusion pressure, causes for hypotension could be investigated if they were not already apparent.

The degree of PCr/Pi depression may also correlate with outcome, if performed on the day of injury, as seen in animal studies (107, 108, 111). However, there is a paucity of outcome data from ^31^P MRS animal studies reporting changes in PCr or Pi performed more than 12–48 h after injury, in what would be a more achievable timeframe clinically. However, as mentioned above, the situation with human TBI patients seems to differ from animal studies, with human TBI causing a higher PCr/ATP or PCr/Pi ratio than healthy controls, and TBI resulting in a more alkaline brain pH when performed 4–21 days after injury. If the Pi peak is not distinguishable from baseline noise, PCr/ATP could be used as an alternative ratio. However, in the event of TBI with hypotension or extreme injury, an equivalent fall in both PCr and ATP species could (in principle) lead to no net change in their relative ratio (PCr/ATP) (102).

^31^P MRS studies performed in the acute to subacute period after injury that display an elevation in the PCr/Pi and PCr/ATP ratios may represent neuroinflammatory changes in TBI (102), which merits further investigation. Further study is ongoing characterizing these changes and their pathophysiological basis.

Although brain free intracellular Mg^2+^ appears to be very sensitive to injury in the acute and subacute period following TBI, it does not easily distinguish between moderate and severe grades of injury. Whereas there may be a greater fall in Mg^2+^ following moderate–severe injury than mild injury, paradoxically there is no change following extreme injury (108).

A summary of the effect of TBI on metabolites interrogated by ^31^P MRS is shown in Table 3.

**Table 3:** Summary of metabolite changes following traumatic brain injury (TBI) detectable with *in vivo* ^31^P MRS.

	Changes in hyperacute stage^a^	Changes in subacute stage	Correlation to prognosis
Metabolites associated with energy metabolism	↓ PCr	↑ PCr/ATP	
	↑ Pi		
	↓ PCr/Pi	↑ PCr/Pi	✓^✽^
Change in pH	↓		✓
Mg^2+^	↓^†^	↑↓	✓^✽^^†^

*PCr, phosphocreatine; Pi, inorganic phosphate; ATP, adenosine triphosphate*.

*↑: increase in metabolite; ↓: decrease in metabolite; ✓: potential clinical use as a prognostic predictor*.

*^✽^Animal studies*.

*^†^In animal studies, Mg^2+^ falls proportionally to injury severity, except for following the most severe TBI*.

## ^13^C MRS

### Hardware and Sensitivity

Whereas *in vivo* ^1^H MRS measures brain metabolites by detecting the hydrogen atoms within these molecules, ^13^C MRS does so by detecting the ^13^C isotopes in their structure. ^13^C MRS is much less sensitive than ^1^H MRS as only 1.1% of naturally occurring carbon is the MR visible isotope ^13^C and most organic molecules contain many more hydrogen atoms than they do carbon atoms. The Larmor (natural) frequency of ^13^C is also a quarter of that of ^1^H, so each atom releases much less energy when it relaxes to be detected by the scanner. These factors combine to give ^13^C MRS a sensitivity of only 0.018% of that of ^1^H MRS (15). Consequently, *in vivo* ^13^C MRS studies are almost always performed with an infusion of ^13^C enriched metabolites to boost the signal from the brain. Even so, large voxels are typically also used to acquire as much signal as possible.

### Methods of Detection, Localization, and Decoupling

The sensitivity of ^13^C can be further improved by various techniques that use the interactions of ^1^Hs naturally bonded to the ^13^C nuclei. Nuclear Overhauser enhancement (NOE) and polarization transfer are two different techniques for increasing signal that transfer some spin polarization from ^1^H to ^13^C. Proton decoupling is another important technique as J-coupling of ^13^C nuclei to their bonded ^1^Hs causes splitting of metabolite peaks into complex patterns of small multiplets that can be difficult to interpret. This interaction can be broken using proton decoupling at the same time as applying either NOE or polarization transfer, further improving spectral resolution (15, 20).

As well as directly detecting the ^13^C in a metabolite, ^13^C MRS can be performed indirectly through detecting the effects of the ^13^C on the ^1^Hs that are bonded to it, termed ^1^H-observe [^13^C-edited] spectroscopy, or proton-observe carbon edited (POCE) spectroscopy. POCE increases the sensitivity even more than using the polarization transfer technique (almost to that of ^1^H MRS) and directly provides ^13^C fractional enrichment values (20). However, this increased sensitivity, comes with the narrow spectral range of the ^1^H MRS scale (5 ppm) where many peaks overlap, so resolving individual peaks can be more difficult. Crowding of the spectra is much less of a problem with the great spectral range of direct ^13^C MRS detection (200 ppm). Also, ^13^C nuclei which do not have ^1^H attached, such as the carboxylate carbon in Glu and Gln can be measured by direct ^13^C MRS while they cannot be measured with the indirect (POCE) MRS technique.

Proton decoupling, NOE, polarization transfer or indirect detection require a ^1^H channel in addition to the ^13^C channel on the RF head coil (20). A complex sequence of pulses must be passed down each channel in quick succession which can result in current induction from one channel to the other, introducing further noise in the spectrum if appropriate arrangement of these coils with effective filtering is not observed.

The RF pulses for broadband proton decoupling deposit a significant amount of energy into the patient, causing tissue heating. The relevance of tissue heating depends on how thermally sensitive the tissue is, and by how much it is heated. The specific absorption rate limit to minimize heating of tissue is critical with regards to the eyes; so ^13^C MRS with proton decoupling is typically performed using surface coils to address ROIs of the brain that avoid them.

Due to the abundance of carbon atoms in the long fatty acid chains of subcutaneous scalp lipids, non-localized ^13^C spectra of the brain are dominated by large lipid peaks at 20–50 ppm. Glycerol that forms their lipid backbone also produces pronounced, but smaller, peaks at 63 and 73 ppm. These scalp peaks must typically be excluded with voxel selection or outer volume suppression for brain metabolites to be measured. Furthermore, the chemical shifts of some key metabolites place them within the lipid range of 20–50 ppm meaning that voxel selection must be rigorous. The high concentration of lipid in cerebral white matter does not pose the same problem as it is bound up tightly in myelin so its MR signal decays too rapidly for detection by *in vivo* ^13^C MRS (128).

### Glycogen

Despite glycogen’s large size [10^6^–10^7^ Da (129)] it is freely mobile and found in the human brain at concentrations of 5 mM/kg in glia (130) so it is the only brain metabolite of interest visible on ^13^C MRS natural abundance studies using reasonable scan times. It is thought to be 100% MR visible (131) with a peak at 100 ppm, split by bonded ^1^Hs if ^13^C spectra are acquired without proton decoupling. Brain glycogen measured by enzymatic extraction has been shown to increase in regions of focal injury after experimental TBI in rats compared to uninjured regions, but it is not known if this correlates with degree of histological injury or outcome (132).

### Dynamic Studies of Glucose, Lactate, Glu, and Gln

Most ^13^C MRS studies of human brain metabolism involve infusion of ^13^C enriched metabolically active substrates and detection of that signal as it makes its way sequentially through different metabolic pools. The most commonly studied substrate in the brain is 1-^13^C glucose: as it is infused it appears in the brain at 94 and 98 ppm (α and β isoforms). It is then metabolized to lactate by glycolysis (principally), producing a lactate peak at 22 ppm in the brain spectra. The ^13^C label is then incorporated into the TCA cycle where it is spun out from α-ketoglutarate as Glu, detectable at 34 ppm. ^13^C Glu is released from neurons and taken up by glia where it is converted into Gln (5), detected at 32 ppm. Using mathematical models and certain assumptions, the rate of brain glucose uptake can be calculated from the appearance of glucose, the rate of glycolysis from the appearance of lactate, the TCA cycle rate from the appearance of Glu and neuronal–astrocyte coupling by the appearance of Gln (5, 15, 133). Alternative labeling patterns of glucose can be used, such as 2-^13^C and U-^13^C6 glucose that share identical biological effects but produce different spectra. There are benefits and limitations for each (20). Dynamic ^13^C studies can also be performed using ^13^C acetate and ^13^C beta-hydroxybutyrate. Acetate is predominantly metabolized by the glia, allowing the metabolic rates of this specific cell population to be measured (5, 20) whereas beta-hydroxybutyrate is predominantly metabolized by neurons during periods of fasting when it supplies 60% of the fuel for brain (5).

Positron emission tomography studies of brain metabolism using [^18^F]-fluorodeoxyglucose can measure the brain’s uptake and phosphorylation of glucose, but are unable to follow its metabolism further downstream: ^13^C MRS measures glucose uptake, but also the TCA cycle rate and neuronal–glial coupling. Changes in the rate of the TCA cycle and Glu/Gln cycling have been reported following stroke, Alzheimer’s disease, and diabetes mellitus (5). No ^13^C infusion studies of human TBI have been reported to date, although the technique has potential to shed light on the effects of TBI on these key processes.

### ^13^C Hyperpolarization

^13^C hyperpolarization is a technique that transiently increases the signal from ^13^C nuclei 10,000-fold (134), allowing detection of ^13^C metabolites in a short timeframe. Without performing hyperpolarization, nuclear polarization is poor because the energy required to align a nuclear spin against a magnetic field is so small that thermal fluctuations can easily overpower these transitions despite using large magnetic fields. Although various hyperpolarization methods exist, the version implemented for clinical studies is dissolution dynamic nuclear polarization (DNP). The following description is from Nelson et al. (135). “Hyperpolarized ^13^C MRI is a relatively new molecular imaging technique with an unprecedented gain in signal intensity of 10,000- to 100,000-fold (134) that can be used to monitor uptake and metabolism of endogenous biomolecules (136, 137). The magnitude of the increase in sensitivity depends on the degree of polarization that is achieved, the T1 relaxation time of the ^13^C agent, the delivery time, and the MR methods applied. Hyperpolarized agents are generated by mixing ^13^C-labeled compounds with an electron paramagnetic agent (EPA), placing them in a 3.35-T magnetic field, cooling to ~1 K, and using microwaves to transfer polarization from the electron spin of the EPA to the ^13^C nuclei of the biomolecule (13). Once the polarization has reached the required level, the sample is rapidly dissolved with hot, sterile water and neutralized to physiological pH, temperature, and osmolarity. Intravenous injection of the hyperpolarized solution and observation using ^13^C MR allow its delivery and metabolic products to be monitored (15). The data must be obtained as rapidly as possible after dissolution because the enhancement decays at a rate determined by the T1 relaxation time of the agent, which is about 60 s for [1-^13^C] pyruvate at 3 T. Translation of hyperpolarized technology into human subjects has been challenging because it requires specialized instrumentation to prepare the agent in a sterile environment, filter out the EPA, perform quality control, and rapidly deliver samples to the patient.” DNP works best for metabolites with carboxylate carbons which have long T1 so polarization decays more slowly; clinical studies typically use [1-^13^C] pyruvate, such as the first-in-human study that interrogated the metabolism of prostate cancer (135). To date, no ^13^C hyperpolarization studies in human TBI brain have been published.

A ^13^C hyperpolarization study of rat TBI has recently been performed by DeVience et al. using 1-^13^C pyruvate (138). Controlled cortical impact of rat brain produced lower ^13^C-bicarbonate signals and higher [1-^13^C] lactate in traumatized regions of brain than non-traumatized brain. This correlated with cortical scarring and persisting cell death on histological analysis performed 30 days after injury. This suggests a shift from oxidative to non-oxidative metabolism due to TBI, in the absence of gross hypoperfusion, as no difference in [1-^13^C] pyruvate signal was seen in the traumatized region. Surprisingly, sham-operated animals that underwent craniotomy, but no intentional cortical injury showed much less significant but similar changes to those exposed to cortical impact. In mice, a ^13^C hyperpolarization study with 1-^13^C pyruvate, performed 12 h after controlled cortical impact to brain showed an increase in the 1-^13^C lactate/1-^13^C pyruvate ratio detected with *in vivo* ^13^C MRS in the injured hemisphere compared to the contralateral uninjured hemisphere (139).

Conventional (non-hyperpolarized) ^13^C MRS studies that rely on the infusion of ^13^C enriched substrates detect downstream metabolites of the substrates infused. Hyperpolarized studies are much more limited due to the very transient nature of hyperpolarization enhancement of ^13^C MRS signal so only metabolites a few steps downstream can be detected before the hyperpolarized effect is lost. Hyperpolarization and conventional ^13^C MRS labeling studies can be considered complementary as they address metabolic pathways on different timescales.

### Summary of ^13^C MRS and Clinical Role

Despite the potential of ^13^C enriched steady-state infusion studies to shed light on the biochemistry of TBI, we do not currently see it as a routine clinical tool in the management of TBI, due to the extensive time required in the scanner for data acquisition, large volumes of expensive ^13^C-labeled infusates required and complex post-acquisition analysis. However, conceivably ^13^C isotope costs may come down in future, scanners become more sensitive, simpler data analysis strategies devised, and workarounds adopted such as starting the infusion outside of the magnet to reduce the time the patient is inside. Other possibilities are natural abundance (unlabeled) studies of brain glycogen that may show changes related to TBI, but few studies to date have demonstrated this and the scan times required are also long (20, 130).

Hyperpolarized ^13^C MRS shows great potential in the monitoring of brain metabolism for the clinical management of TBI. The short acquisition time and clear signal it produces puts it on par with ^1^H MRS, although ^13^C hyperpolarization has the downside of expensive ^13^C-substrate and hyperpolarization equipment, and the larger team of expert staff necessary. Metabolic derangement by elevated lactate/pyruvate or lactate/bicarbonate ratios can be mapped throughout the brain unlike techniques such as microdialysis, which only sample from a single region of brain which may miss key regions where brain energy is failing. As targeted therapies for brain injury become available they may be delivered focally to regions of metabolic dysfunction. *In vivo* ^13^C hyperpolarization is still a relatively new technique so its development with further advances are expected.

## Practical Considerations: MR Conditional Equipment and Risks

Taking critically ill patients with acute severe TBI for an MR study can be challenging; patients typically have multiple monitoring devices and require intensive support. However, with the use of MR conditional ventilators, syringe drivers, and an appropriate ICP monitor setup, a patient’s critical care bed can effectively be recreated inside the MR suite.

Equipment MR compatibility is graded. Whereas plastic ventilator tubing is MR safe at any field strength and is called “MR Safe” a mechanical ventilator may be suitable to use at 3 T but not at 7 T: “MR conditional.” Even if it is designed to be used with a 3 T scanner, often that allows it to be taken into the room, but not right up to the magnet bore where the magnetic field is strongest. Ventilator extension tubing must be prepared to reach the patient.

As well as the projectile risk of ferrous objects, an item’s MR conditional status depends on its performance within the MR environment. Both the changing gradient magnetic fields used for localization and the power and frequency of the RF pulses can cause induction of current in non-ferrous metals. This is greatest when the length of the object, commonly a wire, is a multiple of the wavelength of the RF pulse (140). Furthermore, the ventilator or patient monitor can produce electromagnetic interference that will affect the image or spectra quality.

An important example of this for patients suffering from TBI is the commonly used Codman MircoSensor ICP Transducer (Codman & Shurtleff, Inc.). When using the body (main scanner) coil to transmit and a head coil to detect at 3 T, the electrical current that is induced is sufficient to heat the wire and damage the probe. This necessitates replacement of the probe, and consideration of potential burns to the patient’s skin and brain that are in contact with the wire. This effect can be stopped by looping the extra length of wire away from the patient’s skin, which introduces a radiofrequency choke that limits current induction (141). This allows safe use of the microsensor in a 3 T scanner during MR data acquisition. Two other monitoring devices that are often used in the management of acute severe TBI cannot be used during an MRS study: brain tissue oxygen probes (such as Licox^®^) and microdialysis pumps. Licox catheters must be disconnected with their connecting lead but the attached intracranial probe can generally be left in place for reconnection after the study. Microdialysis catheters may similarly be left attached but the battery that drives the pump is MR unsafe, so must be removed. Whereas these two monitoring systems are useful for clinical management, a brief hiatus is rarely critically disruptive and probably outweighed by the information that MRI and MRS studies provide.

Other specific items that are a projectile risk in the static magnetic field are at risk of current induction causing burning or rotational injury due to changing magnetic fields include: pacemakers and their leads, ECG wires and dots, deep brain and spinal cord stimulation leads, patient oxygen cylinders, some cerebral aneurysm clips, and metal fragments in patients’ eyes. If these are present and non-removable (such as an implanted pacemaker), they will preclude examination by MRS/MRI. In the acute period after a severe TBI, it is difficult exclude a history of a metal fragment in a patient’s eyes, but in practice these would have been detected or excluded on CT examination at presentation for acute TBI. Some tattoos and permanent eyeliners may also be heated by the RF pulses but these are often not an absolute contraindication to examination by MR. The issue of guarding against tissue heating is not just confined to metal fragments but also to uncontaminated tissue and has been mentioned above.

Head coils that completely envelope the head make it difficult accessing the patient’s airway in an emergency. Head coils with joins that can open-up, either hinged along one side, or else with a front half that can be detached completely (see Figure 4B), allow access to the airway in an emergency and make correctly positioning the patient’s head within the head coil easier. This is even more relevant when the patient has prominent intracranial monitoring. A potential obstacle to performing MR studies on patients with acute severe TBI is the lack of head elevation that can be achieved during the scan. This is even more restricted by volume head coils. Head elevation to 30° is an effective initial treatment step in the management of raised ICP (142), but only up to 5° of head elevation can be achieved with padding inside a head coil. Patients with very brittle raised ICP on maximum therapy must wait before an MRS study can be performed if they will not tolerate any period lying flat.

## Summary, Conclusion, and Future Prospects

**Figure 5:**
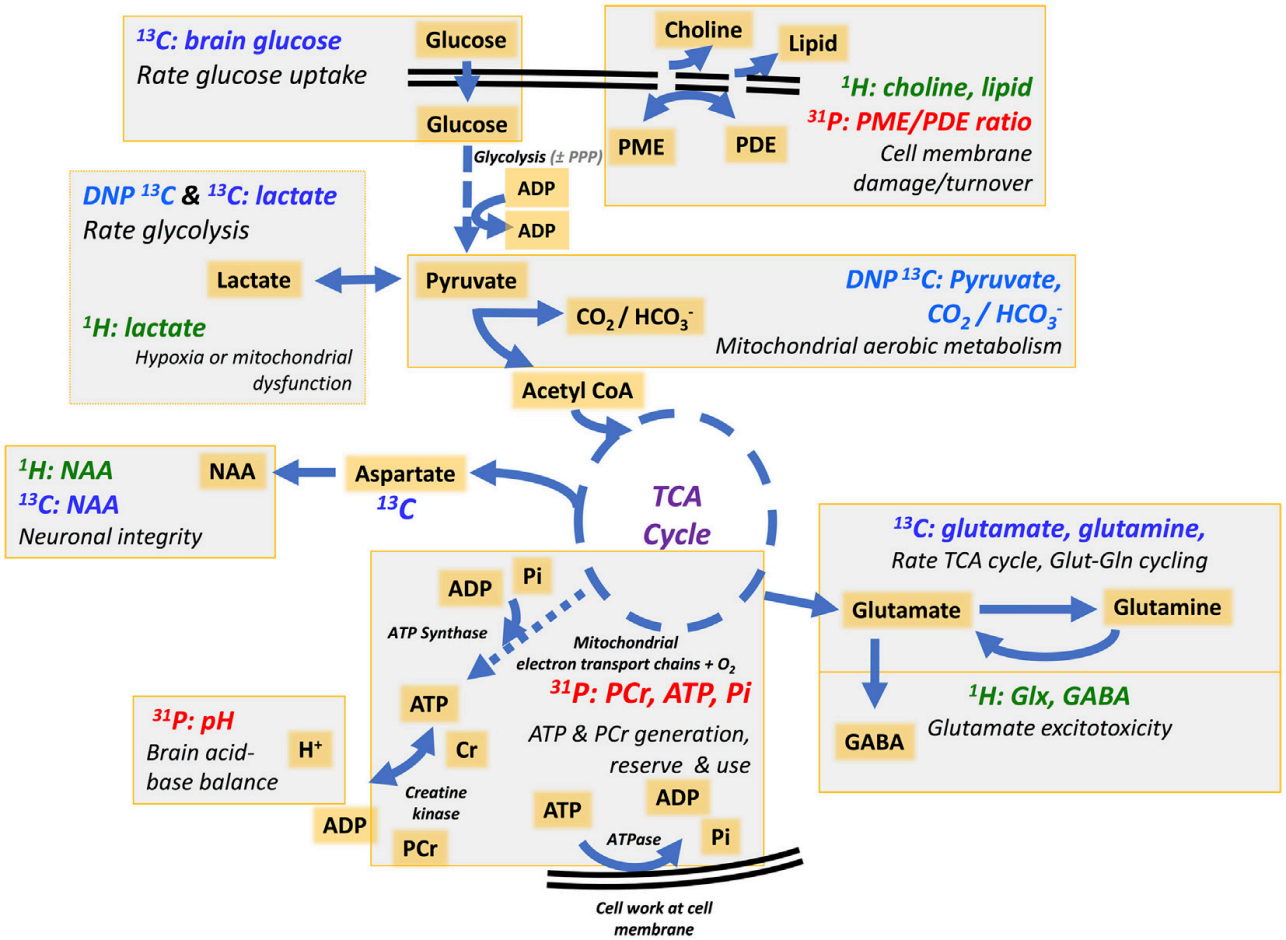
Simplified schematic of different metabolites and processes in the brain that can be interrogated using ^1^H MRS, ^31^P MRS, ^13^C MRS, and DNP ^13^C MRS. ^1^H and ^31^P MRS show endogenous metabolites; ^13^C MRS requires exogenous ^13^C-enriched substrate, while for DNP ^13^C MRS the exogenous ^13^C-enriched substrate is hyperpolarized before administration, transiently boosting ^13^C signal. Pathways include uptake of glucose that is metabolized *via* glycolysis in the cytosol [with a low yield of ATP per mole of glucose consumed] producing pyruvate. Pyruvate can enter mitochondria where it is converted into acetyl-CoA that enters the TCA cycle. Pyruvate remaining in the cytosol can be converted into lactate, simultaneously recycling NADH into NAD^+^ allowing glycolysis to continue. The rate of glucose uptake and glycolysis can be interrogated with ^13^C MRS (glucose and lactate appearance) whereas the relative flux of “anaerobic” metabolism vs. aerobic mitochondrial metabolism can be measured with DNP ^13^C MRS (lactate vs. ${\text{HCO}}_{\text{3}}^{-}$) and ^1^H MRS (lactate). The TCA cycle drives the mitochondrial electron transport chain for high-yield ATP synthesis. The rate of the TCA cycle can be calculated by the rate of appearance of ^13^C labeled glutamate (Glu) (^13^C MRS) and ATP produced measured with ^31^P MRS (γ-ATP, β-ATP, and Pi). Neuronal integrity and mitochondrial function can be measured indirectly by detection of NAA with ^1^H MRS (and ^13^C MRS). Neuronal–glial coupling is represented by Glu–glutamine (Gln) cycling detected by ^13^C MRS, whereas total combined Glu and Gln that may be raised in pathological excitotoxicity can be measured with ^1^H MRS. Cell membrane integrity and damage and turnover may be represented by ^1^H MRS (choline and lipid) and ^31^P MRS (PME/PDE ratio), which also can detect the balance and consumption of high-energy phosphates (ATP, PCr, and Pi). Further details of the above, and other MRS-detectable molecules (including creatine, myoinositol, glycogen, and nicotinamide-adenine dinucleotides), can be found in the text. Abbreviations: ADP, adenosine diphosphate; ATP, adenosine triphosphate; Cr, creatine; DNP, dissolution dynamic nuclear polarization; GABA, gamma-aminobutyric acid; NAA, *N*-acetylaspartate; MRS, magnetic resonance spectroscopy; NAD^+^, nicotinamide adenine dinucleotide oxidized form; NADH, nicotinamide adenine dinucleotide reduced form; PCr, phosphocreatine; PDE, phosphodiester; PME, phosphomonoester; Pi, inorganic phosphate; PPP, pentose phosphate pathway; TCA, tricarboxylic acid.

^1^H, ^31^P, and ^13^C *in vivo* MRS are complementary techniques that allow non-invasive measurement of different aspects of brain metabolism that may contribute to the clinical management of patients with acute severe TBI (see Figure 5).

^13^C MRS measures “upstream” brain energy metabolism: the breakdown of infused ^13^C-labeled glucose (or other sugars) *via* glycolysis and the TCA cycle. To date, few studies of ^13^C MRS in TBI exist, but the development of *in vivo* hyperpolarized techniques shows promise in this field. ^31^P MRS allows measurement of “downstream” metabolism by detecting high-energy phosphates (ATP and PCr) produced by oxidative phosphorylation and creatine kinase in mitochondria. Changes in these metabolites have been noted in a few human and animal studies of TBI but further study is required.

^1^H MRS is the most commonly used MRS technique for studying brain metabolism following TBI. It has the potential to measure various metabolites: some are associated with “upstream” brain energy metabolism such as lactate, Glu and Gln, whose flux can also be measured by ^13^C MRS. Creatine and NAA are associated with the “downstream” metabolism of ATP and PCr, which can also be measured with ^31^P MRS. Free brain lipid and choline are not as directly linked to brain metabolism and are likely markers of cell membrane damage. ^1^H MRS has shown great potential as an additional prognostic tool for patients with acute severe TBI, but the region of the brain that should be studied and how long after injury it should be performed is debatable (143). The development of standardized protocols of acquisition and analysis would facilitate its progression into clinical care.

Magnetic resonance spectroscopy has potential to play a bigger role in Phase II trials of therapies by providing surrogate markers and “tissue fate” measures that can help determine efficacy and inform whether a larger Phase III trial would be worthwhile or not.

Finally, the non-invasive (or minimally invasive) nature of MRS makes it an ideal technique for follow-up of patients’ post-TBI. There is evidence to suggest that TBI produces long-term changes in the brain and that neurodegeneration occurs, with earlier onset of pathologies such as Parkinson’s and Alzheimer’s diseases (144). Better understanding of brain biochemistry may help development of better therapies. MRS is uniquely placed to shed light in such investigations.

## Author Contributions

MS, JLY, and KC designed the review. MS, JLY, KC, AS, and MM drafted the manuscript. All the authors reviewed, edited, and approved the manuscript.

## Conflict of Interest Statement

The authors declare that the research was conducted in the absence of any commercial or financial relationships that could be construed as a potential conflict of interest.

## References

[1] Maas AI Stocchetti N Bullock R. Moderate and severe traumatic brain injury in adults. Lancet Neurol (2008) 7(8):728–41. 10.1016/S1474-4422(08)70164-9 18635021

[2] Carpenter KLH Czosnyka M Jalloh I Newcombe VFJ Helmy A Shannon RJ Systemic, local, and imaging biomarkers of brain injury: more needed, and better use of those already established? Front Neurol (2015) 6:26 10.3389/fneur.2015.00026 25741315 PMC4332345

[3] Timofeev I Carpenter KLH Nortje J Al-Rawi PG O’Connell MT Czosnyka M Cerebral extracellular chemistry and outcome following traumatic brain injury: a microdialysis study of 223 patients. Brain (2011) 134(Pt 2):484–94. 10.1093/brain/awq353 21247930

[4] Carpenter KLH Jalloh I Gallagher CN Grice P Howe DJ Mason A 13C-labelled microdialysis studies of cerebral metabolism in TBI patients. Eur J Pharm Sci (2014) 57:87–97. 10.1016/j.ejps.2013.12.012 24361470 PMC4013834

[5] Rothman DL de Feyter HM de Graaf RA Mason GF Behar KL. 13C MRS studies of neuroenergetics and neurotransmitter cycling in humans. NMR Biomed (2011) 24(8):943–57. 10.1002/nbm.1772 21882281 PMC3651027

[6] Carpenter KLH Jalloh I Hutchinson PJ. Glycolysis and the significance of lactate in traumatic brain injury. Front Neurosci (2015) 9:112. 10.3389/fnins.2015.00112 25904838 PMC4389375

[7] Hutchinson PJ O’Connell MT Seal A Nortje J Timofeev I Al-Rawi PG A combined microdialysis and FDG-PET study of glucose metabolism in head injury. Acta Neurochir (Wien) (2009) 151(1):51–61. 10.1007/s00701-008-0169-1 19099177

[8] Jalloh I Carpenter KLH Grice P Howe DJ Mason A Gallagher CN Glycolysis and the pentose phosphate pathway after human traumatic brain injury: microdialysis studies using 1,2-13C2 glucose. J Cereb Blood Flow Metab (2015) 35(1):111–20. 10.1038/jcbfm.2014.177 25335801 PMC4294402

[9] Glenn TC Martin NA Horning MA McArthur DL Hovda DA Vespa P Lactate: brain fuel in human traumatic brain injury: a comparison with normal healthy control subjects. J Neurotrauma (2015) 32(11):820–32. 10.1089/neu.2014.3483 25594628 PMC4530406

[10] Pellerin L Magistretti PJ. Glutamate uptake into astrocytes stimulates aerobic glycolysis: a mechanism coupling neuronal activity to glucose utilization. Proc Natl Acad Sci U S A (1994) 91(22):10625–9. 10.1073/pnas.91.22.10625 7938003 PMC45074

[11] Jalloh I Carpenter KLH Helmy A Carpenter TA Menon DK Hutchinson PJ. Glucose metabolism following human traumatic brain injury: methods of assessment and pathophysiological findings. Metab Brain Dis (2015) 30(3):615–32. 10.1007/s11011-014-9628-y 25413449 PMC4555200

[12] Williamson DH Lund P Krebs HA. The redox state of free nicotinamide-adenine dinucleotide in the cytoplasm and mitochondria of rat liver. Biochem J (1967) 103(2):514–27. 10.1042/bj1030514 4291787 PMC1270436

[13] Nicholson C Kamali-Zare P Tao L. Brain extracellular space as a diffusion barrier. Comput Vis Sci (2011) 14(7):309–25. 10.1007/s00791-012-0185-9 23172993 PMC3500962

[14] Syková E Nicholson C Diffusion in brain extracellular space. Physiol Rev (2008) 88(4):1277–340. 10.1152/physrev.00027.2007 18923183 PMC2785730

[15] de Graaf RA In Vivo NMR Spectroscopy. 2nd ed Chichester, UK: John Wiley & Sons, Ltd (2007). 55 p.

[16] Hanson LG Is quantum mechanics necessary for understanding magnetic resonance? Concept Magn Reson A (2008) 32A(5):329–40. 10.1002/cmr.a.20123

[17] McRobbie DW Moore EA Graves MJ Prince MR MRI From Picture to Proton. 3rd ed Cambridge: Cambridge University Press (2017).

[18] Brown TR Kincaid BM Ugurbil K. NMR chemical shift imaging in three dimensions. Proc Natl Acad Sci U S A (1982) 79(11):3523–6. 10.1073/pnas.79.11.3523 6954498 PMC346453

[19] van der Graaf M In vivo magnetic resonance spectroscopy: basic methodology and clinical applications. Eur Biophys J (2010) 39(4):527–40. 10.1007/s00249-009-0517-y 19680645 PMC2841275

[20] de Graaf RA Rothman DL Behar KL. State of the art direct 13C and indirect 1H-[13C] NMR spectroscopy in vivo. A practical guide. NMR Biomed (2011) 24(8):958–72. 10.1002/nbm.1761 21919099 PMC3694136

[21] Soher BJ Dale BM Merkle EM. A review of MR physics: 3T versus 1.5T. Magn Reson Imaging Clin N Am (2007) 15(3):277–90. 10.1016/j.mric.2007.06.002 17893049

[22] Vaughan JT Garwood M Collins CM Liu W Delabarre L Adriany G 7T vs. 4T: RF power, homogeneity, and signal-to-noise comparison in head images. Magn Reson Med (2001) 46(1):24–30. 10.1002/mrm.1156 11443707

[23] Marro KI Lee D Shankland EG Mathis CM Hayes CE Friedman SD Quantitative in vivo magnetic resonance spectroscopy using synthetic signal injection. PLoS One (2010) 5(12):e15166. 10.1371/journal.pone.0015166 21203385 PMC3010995

[24] Provencher SW. Automatic quantitation of localized in vivo 1H spectra with LCModel. NMR Biomed (2001) 14(4):260–4. 10.1002/nbm.698 11410943

[25] Naressi A Couturier C Castang I de Beer R Graveron-Demilly D. Java-based graphical user interface for MRUI, a software package for quantitation of in vivo/medical magnetic resonance spectroscopy signals. Comput Biol Med (2001) 31(4):269–86. 10.1016/S0010-4825(01)00006-3 11334636

[26] Naressi A Couturier C Devos JM Janssen M Mangeat C de Beer R Java-based graphical user interface for the MRUI quantitation package. Magma Magn Reson Mater Physics Biol Med (2001) 12(2–3):141–52. 10.1007/BF02668096 11390270

[27] Tedeschi G Righini A Bizzi A Barnett AS Alger JR. Cerebral white matter in the centrum semiovale exhibits a larger N-acetyl signal than does gray matter in long echo time 1H-magnetic resonance spectroscopic imaging. Magn Reson Med (1995) 33(1):127–33. 10.1002/mrm.1910330120 7891527

[28] Croall I Smith FE Blamire AM. Magnetic resonance spectroscopy for traumatic brain injury. Top Magn Reson Imaging (2015) 24(5):267–74. 10.1097/RMR.0000000000000063 26502308

[29] Signoretti S Marmarou A Aygok GA Fatouros PP Portella G Bullock RM. Assessment of mitochondrial impairment in traumatic brain injury using high-resolution proton magnetic resonance spectroscopy. J Neurosurg (2008) 108(1):42–52. 10.3171/JNS/2008/108/01/0042 18173309

[30] Veenith TV Mada M Carter E Grossac J Newcombe V Outtrim J Comparison of inter subject variability and reproducibility of whole brain proton spectroscopy. PLoS One (2014) 9(12):e115304. 10.1371/journal.pone.0115304 25517503 PMC4269459

[31] Ross B Bluml S Magnetic resonance spectroscopy of the human brain. Anat Rec (2001) 265(2):54–84. 10.1002/ar.1058 11323770

[32] Shutter L Tong KA Holshouser BA. Proton MRS in acute traumatic brain injury: role for glutamate/glutamine and choline for outcome prediction. J Neurotrauma (2004) 21(12):1693–705. 10.1089/neu.2004.21.1693 15684761

[33] Mountford CE Stanwell P Lin A Ramadan S Ross B Neurospectroscopy: the past, present and future. Chem Rev (2010) 110(5):3060–86. 10.1021/cr900250y 20387805

[34] Signoretti S Di Pietro V Vagnozzi R Lazzarino G Amorini AM Belli A Transient alterations of creatine, creatine phosphate, N-acetylaspartate and high-energy phosphates after mild traumatic brain injury in the rat. Mol Cell Biochem (2010) 333(1–2):269–77. 10.1007/s11010-009-0228-9 19688182

[35] Gasparovic CC Yeo R Mannell M Ling J Elgie R Phillips J Neurometabolite concentrations in gray and white matter in mild traumatic brain injury: an 1H-magnetic resonance spectroscopy study. J Neurotrauma (2009) 26(10):1635–43. 10.1089/neu.2009-0896 19355814 PMC2822798

[36] Bates TE Strangward M Keelan J Davey GP Munro PM Clark JB. Inhibition of N-acetylaspartate production: implications for 1H MRS studies in vivo. Neuroreport (1996) 7(8):1397–400. 10.1097/00001756-199605310-00014 8856684

[37] Simmons ML Frondoza CG Coyle JT. Immunocytochemical localization of N-acetyl-aspartate with monoclonal antibodies. Neuroscience (1991) 45(1):37–45. 10.1016/0306-4522(91)90101-S 1754068

[38] Moffett JR Ross B Arun P Madhavarao CN Namboodiri AMA. N-Acetylaspartate in the CNS: from neurodiagnostics to neurobiology. Prog Neurobiol (2007) 81(2):89–131. 10.1016/j.pneurobio.2006.12.003 17275978 PMC1919520

[39] Ariyannur PS Moffett JR Manickam P Pattabiraman N Arun P Nitta A Methamphetamine-induced neuronal protein NAT8L is the NAA biosynthetic enzyme: implications for specialized acetyl coenzyme A metabolism in the CNS. Brain Res (2010) 1335:1–13. 10.1016/j.brainres.2010.04.008 20385109

[40] Moffett JR Arun P Ariyannur PS Namboodiri AMA. N-Acetylaspartate reductions in brain injury: Impact on post-injury neuroenergetics, lipid synthesis, and protein acetylation. Front Neuroenergetics (2013) 5:11. 10.3389/fnene.2013.00011 24421768 PMC3872778

[41] Signoretti S Marmarou A Tavazzi B Lazzarino G Beaumont A Vagnozzi R. N-Acetylaspartate reduction as a measure of injury severity and mitochondrial dysfunction following diffuse traumatic brain injury. J Neurotrauma (2001) 18(10):977–91. 10.1089/08977150152693683 11686498

[42] Di Pietro V Amorini A The molecular mechanisms affecting N-acetylaspartate homeostasis following experimental graded traumatic brain injury. Mol Med (2014) 20(1):1 10.2119/molmed.2013.00153 24515258 PMC3966992

[43] Smith DH Cecil KM Meaney DF Chen XH McIntosh TK Gennarelli TA Magnetic resonance spectroscopy of diffuse brain trauma in the pig. J Neurotrauma (1998) 15(9):665–74. 10.1089/neu.1998.15.665 9753214

[44] Condon B Oluoch-Olunya D Hadley D Teasdale G Wagstaff A. Early 1H magnetic resonance spectroscopy of acute head injury: four cases. J Neurotrauma (1998) 15(8):563–71. 10.1089/neu.1998.15.563 9726256

[45] Ross BD Ernst T Kreis R Haseler LJ Bayer S Danielsen E 1H MRS in acute traumatic brain injury. J Magn Reson Imaging (1998) 8(4):829–40. 10.1002/jmri.1880080412 9702884

[46] Cecil KM Hills EC Sandel ME Smith DH McIntosh TK Mannon LJ Proton magnetic resonance spectroscopy for detection of axonal injury in the splenium of the corpus callosum of brain-injured patients. J Neurosurg (1998) 88(5):795–801. 10.3171/jns.1998.88.5.0795 9576245

[47] Holshouser BA Ashwal S Luh GY Shu S Kahlon S Auld KL Proton MR spectroscopy after acute central nervous system injury: outcome prediction in neonates, infants, and children. Radiology (1997) 202(2):487–96. 10.1148/radiology.202.2.9015079 9015079

[48] Marino S Zei E Battaglini M Vittori C Buscalferri A Bramanti P Acute metabolic brain changes following traumatic brain injury and their relevance to clinical severity and outcome. J Neurol Neurosurg Psychiatry (2006) 78(5):501–7. 10.1136/jnnp.2006.099796 17088335 PMC2117835

[49] Garnett MR Blamire AM Corkill RG Cadoux-Hudson TA Rajagopalan B Styles P. Early proton magnetic resonance spectroscopy in normal-appearing brain correlates with outcome in patients following traumatic brain injury. Brain (2000) 123(Pt 1):2046–54. 10.1093/brain/123.10.2046 11004122

[50] Holshouser BA Tong KA Ashwal S Oyoyo U Ghamsary M Saunders D Prospective longitudinal proton magnetic resonance spectroscopic imaging in adult traumatic brain injury. J Magn Reson Imaging (2006) 24(1):33–40. 10.1002/jmri.20607 16755529

[51] Yoon SJ Lee JH Kim ST Chun MH. Evaluation of traumatic brain injured patients in correlation with functional status by localized 1H-MR spectroscopy. Clin Rehabil (2005) 19(2):209–15. 10.1191/0269215505cr813oa 15759537

[52] De Stefano N Matthews PM Arnold DL. Reversible decreases in N-acetylaspartate after acute brain injury. Magn Reson Med (1995) 34(5):721–7. 10.1002/mrm.1910340511 8544693

[53] Friedman SD Brooks WM Jung RE Hart BL Yeo RA. Proton MR spectroscopic findings correspond to neuropsychological function in traumatic brain injury. Am J Neuroradiol (1998) 19(10):1879–85. 9874540 PMC8337737

[54] Shutter L Tong KA Lee A Holshouser BA. Prognostic role of proton magnetic resonance spectroscopy in acute traumatic brain injury. J Head Trauma Rehabil (2006) 21(4):334–49. 10.1097/00001199-200607000-00005 16915009

[55] Wu X Kirov II Gonen O Ge Y Grossman RI Lui YW. MR imaging applications in mild traumatic brain injury: an imaging update. Radiology (2016) 279(3):693–707. 10.1148/radiol.16142535 27183405 PMC4886705

[56] Carpentier A Galanaud D Puybasset L Muller J-C Lescot T Boch A-L Early morphologic and spectroscopic magnetic resonance in severe traumatic brain injuries can detect ‘invisible brain stem damage’ and predict ‘vegetative states’. J Neurotrauma (2006) 23(5):674–85. 10.1089/neu.2006.23.674 16689669

[57] Wild JM Macmillan CS Wardlaw JM Marshall I Cannon J Easton VJ 1H spectroscopic imaging of acute head injury – evidence of diffuse axonal injury. MAGMA (1999) 8(2):109–15. 10.1016/S1352-8661(99)00014-9 10456373

[58] Ariza M Junqué C Mataró M Poca MA Bargalló N Olondo M Neuropsychological correlates of basal ganglia and medial temporal lobe NAA/Cho reductions in traumatic brain injury. Arch Neurol (2004) 61(4):541. 10.1001/archneur.61.4.541 15096403

[59] Faden AI Loane DJ. Chronic neurodegeneration after traumatic brain injury: Alzheimer disease, chronic traumatic encephalopathy, or persistent neuroinflammation? Neurotherapeutics (2015) 12(1):143–50. 10.1007/s13311-014-0319-5 25421001 PMC4322076

[60] Johnson VE Stewart JE Begbie FD Trojanowski JQ Smith DH Stewart W. Inflammation and white matter degeneration persist for years after a single traumatic brain injury. Brain (2013) 136(1):28–42. 10.1093/brain/aws322 23365092 PMC3562078

[61] Helmy A Guilfoyle MR Carpenter KL Pickard JD Menon DK Hutchinson PJ. Recombinant human interleukin-1 receptor antagonist promotes M1 microglia biased cytokines and chemokines following human traumatic brain injury. J Cereb Blood Flow Metab (2016) 36(8):1434–48. 10.1177/0271678X15620204 26661249 PMC4976751

[62] Tran T Ross B Lin A. Magnetic resonance spectroscopy in neurological diagnosis. Neurol Clin (2009) 27(1):21–60,xiii. 10.1016/j.ncl.2008.09.007 19055974

[63] Pascual JM Solivera J Prieto R Barrios L López-Larrubia P Cerdán S Time course of early metabolic changes following diffuse traumatic brain injury in rats as detected by ^1^ H NMR spectroscopy. J Neurotrauma (2007) 24(6):944–59. 10.1089/neu.2006.0190 17600512

[64] Ashwal S Holshouser B Tong K Serna T Osterdock R Gross M Proton spectroscopy detected myoinositol in children with traumatic brain injury. Pediatr Res (2004) 56(4):630–8. 10.1203/01.PDR.0000139928.60530.7D 15295080

[65] Gallagher CN Carpenter KLH Grice P Howe DJ Mason A Timofeev I The human brain utilizes lactate via the tricarboxylic acid cycle: a 13C-labelled microdialysis and high-resolution nuclear magnetic resonance study. Brain (2009) 132(10):2839–49. 10.1093/brain/awp202 19700417

[66] Brown JI Baker AJ Konasiewicz SJ Moulton RJ. Clinical significance of CSF glutamate concentrations following severe traumatic brain injury in humans. J Neurotrauma (1998) 15(4):253–63. 10.1089/neu.1998.15.253 9555971

[67] Bullock R Zauner A Woodward JJ Myseros J Choi SC Ward JD Factors affecting excitatory amino acid release following severe human head injury. J Neurosurg (1998) 89(4):507–18. 10.3171/jns.1998.89.4.0507 9761042

[68] Puts N Edden R In vivo magnetic spectroscopy of GABA: a methodological review. Prog Nucl Magn Spectrosc (2012) 60:1–26. 10.1016/j.pnmrs.2011.06.001 PMC3383792 22293397

[69] Rothman DL Petroff OA Behar KL Mattson RH. Localized 1H NMR measurements of gamma-aminobutyric acid in human brain in vivo. Proc Natl Acad Sci U S A (1993) 90(12):5662–6. 10.1073/pnas.90.12.5662 8516315 PMC46781

[70] Brix MK Ersland L Hugdahl K Dwyer GE Grüner R Noeske R Within- and between-session reproducibility of GABA measurements with MR spectroscopy. J Magn Reson Imaging (2017) 46:421–30. 10.1002/jmri.25588 28205280

[71] Mescher M Merkle H Kirsch J Garwood M Gruetter R Simultaneous in vivo spectral editing and water suppression. NMR Biomed (1998) 11(6):266–72. 10.1002/(SICI)1099-1492(199810)11:6<266::AID-NBM530>3.3.CO;2-A 9802468

[72] Guerriero RM Giza CC Rotenberg A. Glutamate and GABA imbalance following traumatic brain injury. Curr Neurol Neurosci Rep (2015) 15(5):27. 10.1007/s11910-015-0545-1 25796572 PMC4640931

[73] Newcombe VFJ Williams GB Outtrim JG Chatfield D Gulia Abate M Geeraerts T Microstructural basis of contusion expansion in traumatic brain injury: insights from diffusion tensor imaging. J Cereb Blood Flow Metab (2013) 33(6):855–62. 10.1038/jcbfm.2013.11 23423189 PMC3677102

[74] Timofeev I Czosnyka M Carpenter KLH Nortje J Kirkpatrick PJ Al-Rawi PG Interaction between brain chemistry and physiology after traumatic brain injury: impact of autoregulation and microdialysis catheter location. J Neurotrauma (2011) 28(6):849–60. 10.1089/neu.2010.1656 21488707 PMC3113421

[75] Bouzier-Sore A-K Voisin P Canioni P Magistretti PJ Pellerin L. Lactate is a preferential oxidative energy substrate over glucose for neurons in culture. J Cereb Blood Flow Metab (2003) 23:1298–306. 10.1097/01.WCB.0000091761.61714.25 14600437

[76] Patel AB Lai JCK Chowdhury GMI Hyder F Rothman DL Shulman RG Direct evidence for activity-dependent glucose phosphorylation in neurons with implications for the astrocyte-to-neuron lactate shuttle. Proc Natl Acad Sci U S A (2014) 111(14):5385–90. 10.1073/pnas.1403576111 24706914 PMC3986127

[77] López-Villegas D Lenkinski RE Wehrli SL Ho WZ Douglas SD. Lactate production by human monocytes/macrophages determined by proton MR spectroscopy. Magn Reson Med (1995) 34(1):32–8. 10.1002/mrm.1910340107 7674895

[78] Lange T Dydak U Roberts TPL Rowley HA Bjeljac M Boesiger P. Pitfalls in lactate measurements at 3T. AJNR Am J Neuroradiol (2006) 27(4):895–901. 16611787 PMC8133981

[79] Reinstrup P Ståhl N Mellergård P Uski T Ungerstedt U Nordström CH Intracerebral microdialysis in clinical practice: baseline values for chemical markers during wakefulness, anesthesia, and neurosurgery. Neurosurgery (2000) 47(3):701–10. 10.1227/00006123-200009000-00035 10981758

[80] Mcintosh TK Faden AI Bendall MR Vink R Traumatic brain injury in the rat - alterations in brain lactate and Ph as characterized by H-1 and P-31 nuclear-magnetic-resonance. J Neurochem (1987) 49(5):1530–40. 10.1111/j.1471-4159.1987.tb01024.x 3668537

[81] Vink R McIntosh TK Fernyak SE Weiner MW Faden AI Proton and phosphorus NMR studies of traumatic brain injury in rats. Ann N Y Acad Sci (1987) 508(1):497–500. 10.1111/j.1749-6632.1987.tb32948.x

[82] Kirov II Tal A Babb JS Lui YW Grossman RI Gonen O. Diffuse axonal injury in mild traumatic brain injury: a 3D multivoxel proton MR spectroscopy study. J Neurol (2013) 260(1):242–52. 10.1007/s00415-012-6626-z 22886061 PMC3729330

[83] Ashwal S Holshouser BA Shu SK Simmons PL Perkin RM Tomasi LG Predictive value of proton magnetic resonance spectroscopy in pediatric closed head injury. Pediatr Neurol (2000) 23(2):114–25. 10.1016/S0887-8994(00)00176-4 11020636

[84] Aaen GS Holshouser BA Sheridan C Colbert C McKenney M Kido D Magnetic resonance spectroscopy predicts outcomes for children with nonaccidental trauma. Pediatrics (2010) 125(2):295–303. 10.1542/peds.2008-3312 20123781

[85] Makoroff KL Cecil KM Care M Ball WS. Elevated lactate as an early marker of brain injury in inflicted traumatic brain injury. Pediatr Radiol (2005) 35(7):668–76. 10.1007/s00247-005-1441-7 15830194

[86] Hillary FG Liu WC Genova HM Maniker AH Kepler K Greenwald BD Examining lactate in severe TBI using proton magnetic resonance spectroscopy. Brain Inj (2007) 21(9):981–91. 10.1080/02699050701426964 17729050

[87] Haseler LJ Arcinue E Danielsen ER Bluml S Ross BD. Evidence from proton magnetic resonance spectroscopy for a metabolic cascade of neuronal damage in shaken baby syndrome. Pediatrics (1997) 99(1):4–14. 10.1542/peds.99.1.4 8989330

[88] Jalloh I Helmy A Howe DJ Shannon RJ Grice P Mason A Focally perfused succinate potentiates brain metabolism in head injury patients. J Cereb Blood Flow Metab (2017) 37(7):2626–38. 10.1177/0271678X16672665 27798266 PMC5482384

[89] Golding EM Dobson GP Golding RM. A critical assessment of noise-induced errors in 31P MRS: application to the measurement of free intracellular magnesium in vivo. Magn Reson Med (1996) 35(2):174–85. 10.1002/mrm.1910350208 8622581

[90] Barker PB Butterworth EJ Boska MD Nelson J Welch KM Magnesium and pH imaging of the human brain at 3.0 Tesla. Magn Reson Med (1999) 41(2):400–6. 10.1002/(SICI)1522-2594(199902)41:2<400::AID-MRM26>3.0.CO;2-E 10080290

[91] Prichard JW Shulman RG NMR spectroscopy of brain metabolism in vivo. Annu Rev Neurosci (1986) 9:61–85. 10.1146/annurev.ne.09.030186.000425 2871798

[92] Andrade CS Otaduy MCG Park EJ Leite CC Phosphorus-31 MR spectroscopy of the human brain. Int J Cur Res Rev (2014) 6(9):41–57.

[93] Zhu X-H Qiao H Du F Xiong Q Liu X Zhang X Quantitative imaging of energy expenditure in human brain. Neuroimage (2012) 60(4):2107–17. 10.1016/j.neuroimage.2012.02.013 22487547 PMC3325488

[94] Erecinska M Silver IA ATP and brain function. J Cereb Blood Flow Metab (1989) 9:2–19. 10.1038/jcbfm.1989.2 2642915

[95] Komoroski RA Pearce JM Mrak RE. 31P NMR spectroscopy of phospholipid metabolites in postmortem schizophrenic brain. Magn Reson Med (2008) 59(3):469–74. 10.1002/mrm.21516 18306399

[96] Yoshizaki K Watari H Radda GK. Role of phosphocreatine in energy transport in skeletal muscle of bullfrog studied by 31P-NMR. Biochim Biophys Acta (1990) 1051(2):144–50. 10.1016/0167-4889(90)90186-H 2310769

[97] Jacobus WE. Theoretical support for the heart phosphocreatine energy transport shuttle based on the intracellular diffusion limited mobility of ADP. Biochem Biophys Res Commun (1985) 133(3):1035–41. 10.1016/0006-291X(85)91240-9 4084301

[98] Chance B Eleff S Leigh JS. Noninvasive, nondestructive approaches to cell bioenergetics. Proc Natl Acad Sci U S A (1980) 77(12):7430–4. 10.1073/pnas.77.12.7430 6938983 PMC350517

[99] Wallimann T Wyss M Brdiczka D Nicolay K Eppenberger HM. Intracellular compartmentation, structure and function of creatine kinase isoenzymes in tissues with high and fluctuating energy demands: the ‘phosphocreatine circuit’ for cellular energy homeostasis. Biochem J (1992) 281(Pt 1):21–40. 1731757 10.1042/bj2810021 PMC1130636

[100] Mason GF Petersen KF De Graaf RA Kanamatsu T Otsuki T Rothman DL A comparison of 13C NMR measurements of the rates of glutamine synthesis and the tricarboxylic acid cycle during oral and intravenous administration of [1-13C]glucose. Brain Res Protoc (2003) 10:181–90. 10.1016/S1385-299X(02)00217-9 12565689

[101] Hetherington HP Spencer DD Vaughan JT Pan JW Quantitative 31P spectroscopic imaging of human brain at 4 Tesla: assessment of gray and white matter differences of phosphocreatine and ATP. Magn Reson Med (2001) 45(1):46–52. 10.1002/1522-2594(200101)45:1<46::AID-MRM1008>3.0.CO;2-N 11146485

[102] Garnett MR Corkill RG Blamire AM Rajagopalan B Manners DN Young JD Altered cellular metabolism following traumatic brain injury: a magnetic resonance spectroscopy study. J Neurotrauma (2001) 18(3):231–40. 10.1089/08977150151070838 11284544

[103] Ishige N Pitts LH Berry I Nishimura MC James TL. The effects of hypovolemic hypotension on high-energy phosphate metabolism of traumatized brain in rats. J Neurosurg (1988) 68(1):129–36. 10.3171/jns.1988.68.1.0129 3335898

[104] Vink R McIntosh TK Weiner MW Faden AI. Effects of traumatic brain injury on cerebral high-energy phosphates and pH: a 31P magnetic resonance spectroscopy study. J Cereb Blood Flow Metab (1987) 7(5):563–71. 10.1038/jcbfm.1987.106 3654796

[105] Golding EM Golding RM. Interpretation of 31P MRS spectra in determining intracellular free magnesium and potassium ion concentrations. Magn Reson Med (1995) 33(4):467–74. 10.1002/mrm.1910330403 7776876

[106] Ren J Sherry AD Malloy CR 31 P-MRS of healthy human brain: ATP synthesis, metabolite concentrations, pH, and T 1 relaxation times. NMR Biomed (2015) 28(11):1455–62. 10.1002/nbm.3384 26404723 PMC4772768

[107] Vink R McIntosh TK Yamakami I Faden AI. 31P NMR characterization of graded traumatic brain injury in rats. Magn Reson Med (1988) 6(1):37–48. 10.1002/mrm.1910060105 3352504

[108] Vink R Faden AI McIntosh TK. Changes in cellular bioenergetic state following graded traumatic brain injury in rats: determination by phosphorus 31 magnetic resonance spectroscopy. J Neurotrauma (1988) 5(4):315–30. 10.1089/neu.1988.5.315 3249310

[109] Sauter A Rudin M. Determination of creatine kinase kinetic parameters in rat brain by NMR magnetization transfer: Correlation with brain function. J Biol Chem (1993) 268(18):13166–71. 8514755

[110] Cernak I Vink R Zapple DN Cruz MI Ahmed F Chang T The pathobiology of moderate diffuse traumatic brain injury as identified using a new experimental model of injury in rats. Neurobiol Dis (2004) 17(1):29–43. 10.1016/j.nbd.2004.05.011 15350963

[111] Vink R McIntosh TKK Demediuk P Weiner MW Faden AI. Decline in intracellular free Mg2+ is associated with irreversible tissue injury after brain trauma. J Biol Chem (1988) 263(2):757–61. 3335524

[112] Suzuki M Nishina M Endo M Matsushita K Tetsuka M Shima K Decrease in cerebral free magnesium concentration following closed head injury and effects of VA-045 in rats. Gen Pharmacol (1997) 28(1):119–21. 10.1016/S0306-3623(96)00148-6 9112087

[113] Gupta AK Zygun DA Johnston AJ Steiner LA Al-Rawi PG Chatfield D Extracellular brain pH and outcome following severe traumatic brain injury. J Neurotrauma (2004) 21(6):678–84. 10.1089/0897715041269722 15253796

[114] Timofeev I Nortje J Al-Rawi PG Hutchinson PJA Gupta AK. Extracellular brain pH with or without hypoxia is a marker of profound metabolic derangement and increased mortality after traumatic brain injury. J Cereb Blood Flow Metab (2013) 33(3):422–7. 10.1038/jcbfm.2012.186 23232949 PMC3587815

[115] Puri BK Treasaden IH. A human in vivo study of the extent to which 31-phosphorus neurospectroscopy phosphomonoesters index cerebral cell membrane phospholipid anabolism. Prostaglandins Leukot Essent Fatty Acids (2009) 81(5–6):307–8. 10.1016/j.plefa.2009.10.003 19906517

[116] Mason GF Chu W-J Vaughan JT Ponder SL Twieg DB Adams D Evaluation of 31P metabolite differences in human cerebral gray and white matter. Magn Reson Med (1998) 39(3):346–53. 10.1002/mrm.1910390303 9498589

[117] Forester BP Berlow YA Harper DG Jensen JE Lange N Froimowitz MP Age-related changes in brain energetics and phospholipid metabolism. NMR Biomed (2010) 23(3):242–50. 19908224 10.1002/nbm.1444

[118] Schmoller A Hass T Strugovshchikova O Melchert UH Scholand-Engler HG Peters A Evidence for a relationship between body mass and energy metabolism in the human brain. J Cereb Blood Flow Metab (2010) 30(7):1403–10. 10.1038/jcbfm.2010.48 20389303 PMC2949217

[119] Nilsson L Siesjö BK Influence of anaesthetics on the balance between production and utilization of energy in the brain. J Neurochem (1974) 23(1):29–36. 10.1111/j.1471-4159.1974.tb06912.x 4852427

[120] Forsen S Hoffman RA Study of moderately rapid chemical exchange reactions by means of nuclear magnetic double resonance. J Chem Phys (1963) 39(11):2892–901. 10.1063/1.1734121

[121] Jeong EK Sung YH Kim SE Zuo C Shi X Mellon EA Measurement of creatine kinase reaction rate in human brain using magnetization transfer image-selected in vivo spectroscopy (MT-ISIS) and a volume 31P/1H radiofrequency coil in a clinical 3-T MRI system. NMR Biomed (2011) 24(7):765–70. 10.1002/nbm.1636 21834000 PMC3143248

[122] Chen W Zhu XH Adriany G Ugurbil K. Increase of creatine kinase activity in the visual cortex of human brain during visual stimulation: a 31P magnetization transfer study. Magn Reson Med (1997) 38(4):551–7. 10.1002/mrm.1910380408 9324321

[123] Kemp GJ Brindle KM. What do magnetic resonance-based measurements of Pi→ATP flux tell us about skeletal muscle metabolism? Diabetes (2012) 61(8):1927–34. 10.2337/db11-1725 22826313 PMC3402329

[124] Befroy DE Rothman DL Petersen KF Shulman GI. 31P-magnetization transfer magnetic resonance spectroscopy measurements of in vivo metabolism. Diabetes (2012) 61(11):2669–2678. 10.2337/db12-0558 23093656 PMC3478545

[125] From AHL Ugurbil K Standard magnetic resonance-based measurements of the Pi→ATP rate do not index the rate of oxidative phosphorylation in cardiac and skeletal muscles. AJP Cell Physiol (2011) 301(1):C1–11. 10.1152/ajpcell.00345.2010 PMC3129822 21368294

[126] Sleigh A Savage DB Williams GB Porter D Carpenter TA Brindle KM ^31^ P magnetization transfer measurements of P_i_ → ATP flux in exercising human muscle. J Appl Physiol (2016) 120(6):649–56. 10.1152/japplphysiol.00871.2015 26744504 PMC4796179

[127] Du F Zhu X-H Zhang Y Friedman M Zhang N Ugurbil K Tightly coupled brain activity and cerebral ATP metabolic rate. Proc Natl Acad Sci U S A (2008) 105(17):6409–14. 10.1073/pnas.0710766105 18443293 PMC2359810

[128] Wilhelm MJ Ong HH Wehrli SL Li C Tsai P-H Hackney DB Direct magnetic resonance detection of myelin and prospects for quantitative imaging of myelin density. Proc Natl Acad Sci U S A (2012) 109(24):9605–10. 10.1073/pnas.1115107109 22628562 PMC3386098

[129] Wishart DS Jewison T Guo AC Wilson M Knox C Liu Y HMDB 3.0 – the human metabolome database in 2013. Nucleic Acids Res (2013) 41(D1):D801–7. 10.1093/nar/gks1065 23161693 PMC3531200

[130] Öz G Henry P-G Seaquist ER Gruetter R. Direct, noninvasive measurement of brain glycogen metabolism in humans. Neurochem Int (2003) 43(4–5):323–9. 10.1016/S0197-0186(03)00019-6 12742076

[131] Sillerud LO Shulman RG. Structure and metabolism of mammalian liver glycogen monitored by carbon-13 nuclear magnetic resonance. Biochemistry (1983) 22(5):1087–94. 10.1021/bi00274a015 6838841

[132] Otori T Friedland JC Sinson G McIntosh TK Raghupathi R Welsh FA. Traumatic brain injury elevates glycogen and induces tolerance to ischemia in rat brain. J Neurotrauma (2004) 21(6):707–18. 10.1089/0897715041269623 15253799

[133] Cheshkov S Dimitrov IE Jakkamsetti V Good L Kelly D Rajasekaran K Oxidation of [U-^13^ C]glucose in the human brain at 7T under steady state conditions. Magn Reson Med (2017). 10.1002/mrm.26603 28112825 PMC5522773

[134] Ardenkjaer-Larsen JH Fridlund B Gram A Hansson G Hansson L Lerche MH Increase in signal-to-noise ratio of >10,000 times in liquid-state NMR. Proc Natl Acad Sci U S A (2003) 100(18):10158–63. 10.1073/pnas.1733835100 12930897 PMC193532

[135] Nelson SJ Kurhanewicz J Vigneron DB Larson PEZ Harzstark AL Ferrone M Metabolic imaging of patients with prostate cancer using hyperpolarized [1-13 C] pyruvate. Sci Transl Med (2013) 5(198):198ra108 10.1126/scitranslmed.3006070 PMC4201045 23946197

[136] Golman K Zandt R Thaning M. Real-time metabolic imaging. Proc Natl Acad Sci U S A (2006) 103(30):11270–5. 10.1073/pnas.0601319103 16837573 PMC1544077

[137] Golman K Petersson JS Metabolic imaging and other applications of hyperpolarized 13C1. Acad Radiol (2006) 13(8):932–42. 10.1016/j.acra.2006.06.001 16843845

[138] DeVience SJ Lu X Proctor J Rangghran P Melhem ER Gullapalli R Metabolic imaging of energy metabolism in traumatic brain injury using hyperpolarized [1-13C]pyruvate. Sci Rep (2017) 7(1):1907 10.1038/s41598-017-01736-x 28507314 PMC5432492

[139] Guglielmetti C Najac AC Van der linden A Ronen SM Rose S Chaumeil MM MR metabolic imaging of neuroinflammation using HP 13C MR. 2017 HMTRC Workshop, San Francisco: Hyperpolarized MRI Technology Resource Center, Department of Radiology & Biomedical Imaging, University of California (2017).

[140] Shellock FG MRISafety.com (2017). Available from: http://mrisafety.com/

[141] Newcombe VFJ Hawkes RC Harding SG Willcox R Brock S Hutchinson PJ Potential heating caused by intraparenchymal intracranial pressure transducers in a 3-tesla magnetic resonance imaging system using a body radiofrequency resonator: assessment of the Codman MicroSensor transducer. J Neurosurg (2008) 109(1):159–64. 10.3171/JNS/2008/109/7/0159 18590450

[142] Feldman Z Kanter MJ Robertson CS Contant CF Hayes C Sheinberg MA Effect of head elevation on intracranial pressure, cerebral perfusion pressure, and cerebral blood flow in head-injured patients. J Neurosurg (1992) 76(2):207–11. 10.3171/jns.1992.76.2.0207 1730949

[143] Marino S Ciurleo R Bramanti P Federico A De Stefano N. 1H-MR spectroscopy in traumatic brain injury. Neurocrit Care (2011) 14(1):127–33. 10.1007/s12028-010-9406-6 20737247

[144] Green REA Brain injury as a neurodegenerative disorder. Front Hum Neurosci (2016) 9:615 10.3389/fnhum.2015.00615 26778994 PMC4700280

[145] Lei H Ugurbil K Chen W Measurement of unidirectional Pi to ATP flux in human visual cortex at 7 T by using in vivo 31P magnetic resonance spectroscopy. Proc Natl Acad Sci U S A (2003) 100(24):14409–14. 10.1073/pnas.2332656100 14612566 PMC283605

