# Corrigendum: Assessing Metabolism and Injury in Acute Human Traumatic Brain Injury with Magnetic Resonance Spectroscopy: Current and Future Applications

**DOI:** 10.3389/fneur.2017.00642

**Published:** 2017-12-01

**Authors:** Matthew G. Stovell, Jiun-Lin Yan, Alison Sleigh, Marius O. Mada, T. Adrian Carpenter, Peter J. A. Hutchinson, Keri L. H. Carpenter

**Affiliations:** ^1^Division of Neurosurgery, Department of Clinical Neurosciences, University of Cambridge, Cambridge, United Kingdom; ^2^Department of Neurosurgery, Keelung Chang Gung Memorial Hospital, Chang Gung University College of Medicine, Taoyuan, Taiwan; ^3^Wolfson Brain Imaging Centre, Department of Clinical Neurosciences, University of Cambridge, Cambridge, United Kingdom; ^4^National Institute for Health Research/Wellcome Trust Clinical Research Facility, Cambridge University Hospitals NHS Foundation Trust, Cambridge, United Kingdom

**Keywords:** ^1^H MRS, ^31^P MRS, ^13^C MRS, trauma, traumatic brain injury, energy metabolism, biomarker

**Missing Funding**

In the original article, we neglected to include the funder Medical Research Council, Grant No. G1002277, ID98489 for this study. We neglected to include the Medical Research Council in our list of funders in the Acknowledgements section. The authors apologize for this error and state that this does not change the scientific conclusions of the article in any way.

**Text Correction**

In the original article, there was an error. We neglected to include the Medical Research Council in our list of funders in the Acknowledgements section.

A correction has been made to the Acknowledgements, page 17:

The authors thank T. V. Veenith, P. J. Coles, and D. K. Menon for providing images for Figure 2. The authors also thank D. K. Menon for helpful comments. Our cerebral metabolism studies were funded by the Medical Research Council (Grant No. G1002277, ID98489). The following funding sources should also be acknowledged: PH is supported by a National Institute for Health Research (NIHR) Research Professorship, Academy of Medical Sciences/Health Foundation Senior Surgical Scientist Fellowship and the National Institute for Health Research Biomedical Research Centre, Cambridge. PH and KC are supported by the NIHR Biomedical Research Centre, Cambridge. MS is supported by PH’s NIHR Research Professorship. AS is funded by the NIHR *via* an award to the Cambridge NIHR/Wellcome Trust Clinical Research Facility. MM is funded by the Medical Research Council. The funders had no role in study design, data collection and analysis, decision to publish, or preparation of the manuscript.

## Conflict of Interest Statement

The authors declare that the research was conducted in the absence of any commercial or financial relationships that could be construed as a potential conflict of interest.

